# Supplementary material for: Association between sleep duration and chronic lung diseases among Chinese middle-aged and older adults: A cross-sectional study
Source: PLoS One. 2026 Mar 19;21(3):e0337482. doi: 10.1371/journal.pone.0337482 (PMC13001977; doi:10.1371/journal.pone.0337482)
Supplement: Attachment — Submitted filename: Comments_CLD.docx [file pone.0337482.s004.docx]

This study examined the association between sleep duration and chronic lung diseases among Chinese middle-aged and older adults: a cross-sectional study

**# Major comments**

It is not relevant to add source of their data in the introduction section.

Section 2.1. The author did not clearly explained how often do the survey is done (is this a yearly survey, per 6 months or…), I also missed how could the survey done, online? Please explain.

Section 2.5: “The Kruskal-Wallis test, along with Chi-square or Fisher’s exact tests, is utilized to compare the covariates”

It is not entirely clear what does the author means here, it is not clear what covariates comparison is done and why need to do that, probably if the idea is to select covariates those are most associated with the outcome, for example based on chi-square association, I will not suggest this at all. Some covariates are typically confounders to other, so, dropping those covariates based on Kruskal-Wallis test, Chi-square or Fisher’s exact tests may lead to bias results. I have also checked the tables added at the end of the manuscript, however, I did not find any results that author attached to this manuscript, I suggest to remove tests that are not used.

“We assessed all covariates for collinearity using the variance inflation factor (VIF) and excluded those with VIF values exceeding 5, as they were deemed collinear”

Any reference for the cutoff?

It is not clear why the author a three-piecewise linear regression model selected other than models that can incorporate either the three categories like multinomial or other ordinal model, at first, it is not even very clear why the author want to classify the sleeping time. For a potential non-linearity, there are models that can incorporate the non-linearity including generalized-additive model.

Can the authors provide more explanation on how the inflection points at seven and eight hours were determined? How sensitive are these results to different model specifications?

**Result**

I have read that longer sleep duration (>8 hours) was not significantly associated with CLDs. For this might rather due to low power, I suggest the author to check for insufficient power specifically for this sleeping to group. Recalling my previous comment, categorizing variables, sometimes in non-systematic way may lead to potential under power.

**Discussion**

Overall, I strongly suggest the author to revise the discussion. The discussion should start with the main findings of this study, and then proceed to discussing each important association in detail. What I missed a lot is that the author did not mention important parts about the literature they cited, for example what data they used, is that cross-sectional, cohort or….design, what years did the study covered, which population group, those are main things to mention, otherwise, there is no way for readers to know whether their study is comparative to the literature they cited or not. It is also important to mention whether their findings is in agreement with the literature they cited other than just discussing the literature.

Finally, I missed the added values of this study. The authors explained quite will in the introduction that there are much study on the same area, however, the added values of this study is not well explained.
