# Supplementary material for: Association between sleep duration and chronic lung diseases among Chinese middle-aged and older adults: A cross-sectional study
Source: PLoS One. 2026 Mar 19;21(3):e0337482. doi: 10.1371/journal.pone.0337482 (PMC13001977; doi:10.1371/journal.pone.0337482)
Supplement: Attachment — Submitted filename: Response to Reviewers.docx [file pone.0337482.s005.docx]

We Prof. Gebrekidan Ewnetu Tarekegn for the advice on our manuscript. We also thank the reviewers for the comments and suggestions. Based on the suggestions, we have made revisions to the relevant parts of the manuscript. The main modified part is marked in yellow/green in the paper. The main corrections in the paper and the response to academic editor and the reviewer's comments are as follows.

**Academic editor:**

1. A rebuttal letter that responds to each point raised by the academic editor and reviewer(s). You should upload this letter as a separate file labeled 'Response to Reviewers'.

We greatly appreciate your reminder. The necessary revisions have been completed and upload.

2. A marked-up copy of your manuscript that highlights changes made to the original version. You should upload this as a separate file labeled 'Revised Manuscript with Track Changes'.

We greatly appreciate your reminder. The necessary revisions have been completed and upload.

3. An unmarked version of your revised paper without tracked changes. You should upload this as a separate file labeled 'Manuscript'.

We greatly appreciate your reminder. The necessary revisions have been completed and upload.

4. If you would like to make changes to your financial disclosure, please include your updated statement in your cover letter. Guidelines for resubmitting your figure files are available below the reviewer comments at the end of this letter.

We greatly appreciate your reminder. No financial disclosure changed.

5. Please ensure that your manuscript meets PLOS ONE's style requirements, including those for file naming.

We greatly appreciate your reminder. The necessary revisions have been completed and upload.

6. In the online submission form you indicate that your data is not available for proprietary reasons and have provided a contact point for accessing this data. Please note that your current contact point is a co-author on this manuscript. According to our Data Policy, the contact point must not be an author on the manuscript and must be an institutional contact, ideally not an individual. Please revise your data statement to a non-author institutional point of contact, such as a data access or ethics committee, and send this to us via return email. Please also include contact information for the third party organization, and please include the full citation of where the data can be found.

Thank you for raising this important point about data accessibility. We have revised our data availability statement in our revised manuscript in Data availability section, highlighted in yellow, as follows: The raw data for this study is available from: http://charls.pku.edu.cn/en. The dataset generated from this study can be obtained from the supporting information.

7. PLOS requires an ORCID iD for the corresponding author in Editorial Manager on papers submitted after December 6th, 2016. Please ensure that you have an ORCID iD and that it is validated in Editorial Manager. To do this, go to ‘Update my Information’ (in the upper left-hand corner of the main menu), and click on the Fetch/Validate link next to the ORCID field. This will take you to the ORCID site and allow you to create a new iD or authenticate a pre-existing iD in Editorial Manager.

Thank you very much for your reminder. We have applied for and implemented the ORCID ID.

8. Your ethics statement should only appear in the Methods section of your manuscript. If your ethics statement is written in any section besides the Methods, please move it to the Methods section and delete it from any other section. Please ensure that your ethics statement is included in your manuscript, as the ethics statement entered into the online submission form will not be published alongside your manuscript.

Thank you very much for your suggestion. The position of ethics statement has been adjusted to the Methods section.

9. Please include a separate caption for each figure in your manuscript.

Thank you very much for your suggestion. This has been added to the manuscript.

10. Please include your tables as part of your main manuscript and remove the individual files. Please note that supplementary tables (should remain/ be uploaded) as separate "supporting information" files.

Thank you very much for your reminder. The tables has been removed and the "supporting information" files has been uploaded.

10. We noticed you have some minor occurrence of overlapping text with the previous publication(s), which needs to be addressed:

Thank you very much for your reminder. The overlapping information has been revised.

11. While revising your submission, please upload your figure files to the Preflight Analysis and Conversion Engine (PACE) digital diagnostic tool, https://pacev2.apexcovantage.com/. PACE helps ensure that figures meet PLOS requirements. To use PACE, you must first register as a user. Registration is free. Then, login and navigate to the UPLOAD tab, where you will find detailed instructions on how to use the tool. If you encounter any issues or have any questions when using PACE, please email PLOS at figures@plos.org. Please note that Supporting Information files do not need this step.

Thank you very much for your reminder. Despite multiple attempts to upload the images to the website, we have not received any results. However, we have adjusted the image format to .TIFF with a resolution above 300 dpi in accordance with the journal's requirements.

**Reviewer #1:**

1. **It is not relevant to add source of their data in the introduction section.**

Thank you for your valuable feedback. We agree that mentioning the data source in the Introduction section may not be appropriate. Following your suggestion, we have removed the mention of the data source from this section. We have marked the context of revision in green in the final paragraph of the Introduction section.

**2. Section 2.1. The author did not clearly explained how often do the survey is done (is this a yearly survey, per 6 months or…), I also missed how could the survey done, online? Please explain.**

Thank you for your valuable feedback. We provided a detailed introduction to the basic information of CHARLS, mainly including the tracking survey design, the surveyed population, the sampling method, the follow-up interval, and the survey content. We have marked the context of revision **in green in the first paragraph of the Methods section.**

3. **Section 2.5: “The Kruskal-Wallis test, along with Chi-square or Fisher’s exact tests, is utilized to compare the covariates”. It is not entirely clear what does the author means here, it is not clear what covariates comparison is done and why need to do that, probably if the idea is to select covariates those are most associated with the outcome, for example based on chi-square association, I will not suggest this at all. Some covariates are typically confounders to other, so, dropping those covariates based on Kruskal-Wallis test, Chi-square or Fisher’s exact tests may lead to bias results. I have also checked the tables added at the end of the manuscript, however, I did not find any results that author attached to this manuscript, I suggest to remove tests that are not used.**

Thank you very much for your meticulous observations. The previous description was not clear enough. Therefore, we re-conducted the normality test of the data to determine the appropriate methods for group comparisons. We have also made revisions in the first paragraph of the statistical analysis section.

Software analysis results:

The age variable was non-normally distributed:

4. **“We assessed all covariates for collinearity using the variance inflation factor (VIF) and excluded those with VIF values exceeding 5, as they were deemed collinear”.Any reference for the cutoff?**

Thank you very much for your suggestions. We referred to the relevant literature (Song, Y., H. Liu and Y. Liu, The association between nap time, nighttime sleep and depression in Chinese older adults: A cross-sectional study. PLoS One, 2024. 19(6): p. e0302939.), and defined this cut off as 10, and revised this context in the second paragraph of the Statistical analysis section and the second paragraph of the Results section. Marked in green.

5. **It is not clear why the author a three-piecewise linear regression model selected other than models that can incorporate either the three categories like multinomial or other ordinal model, at first, it is not even very clear why the author want to classify the sleeping time. For a potential non-linearity, there are models that can incorporate the non-linearity including generalized-additive model. Can the authors provide more explanation on how the inflection points at seven and eight hours were determined? How sensitive are these results to different model specifications?**

Thank you very much for your detailed observation. Representing it with a three-segment linear regression model is not very accurate. We referred to several articles and modified it to the two-piecewise regression model (	Li, W., et al., A novel U-shaped relationship between BMI and risk of generalized aggressive periodontitis in Chinese: A cross-sectional study. J Periodontol, 2019. 90(1): p. 82-89; Chen, C. and J.L. Dai, Triglyceride to high-density lipoprotein cholesterol (HDL-C) ratio and arterial stiffness in Japanese population: a secondary analysis based on a cross-sectional study. Lipids Health Dis, 2018. 17(1): p. 130.). Besides, as you mentioned, we also used generalized additive model with fitting smoothness to identify the non-linear relationship.

We referred to relevant literature (Ruan, Z., et al., The association between sleep duration, respiratory symptoms, asthma, and COPD in adults. Front Med (Lausanne), 2023. 10: p. 1108663;	Wang, S., et al., Sleep duration and its association with demographics, lifestyle factors, poor mental health and chronic diseases in older Chinese adults. Psychiatry Research, 2017. 257: p. 212-218.), categorized sleep duration to describe the characteristics of the three groups, and selected 7 and 8 as the two inflection points based on the curve fitting graph. Log-likelihood ratio test comparing one-line (non-segmented) model to segmented regression model was used to determine whether threshold exists.

In addition, we calculated the number of missing cases and performed multiple imputation. The specific results can be found in the Supporting information (S3 Table, S4 Table). Analysis of the imputed data yielded consistent results, indicating that shorter sleep duration was associated with a higher prevalence of chronic lung disease, and the turning points at 7 and 8 hours remained exist. However, considering that our study population consists of middle-aged and elderly Chinese individuals, extrapolation beyond the inflection point should be approached with caution, which we have addressed in the limitations section.

The original text has also been revised in the second paragraph of the Statistical analysis section, in the third paragraph of the Resulds section, in the second paragraph of the Sensitivity analyses, the last Limitations section, and marked with green.

| S3 Table. Logistic regression analysis between sleep duration and chronic lung diseases. | | | | | | | | | |  |
| --- | --- | --- | --- | --- | --- | --- | --- | --- | --- | --- |
| Variables | Model 1 | P | Model 2 | | P | Model 3 | | | P |  |
|  | OR (95% CI) |  | OR (95% CI) | |  | OR (95% CI) | | |  |  |
| Sleep duration | 0.93 (0.90, 0.95) | < 0.001 | 0.93 (0.91, 0.96) | | < 0.001 | 0.96 (0.93, 0.99) | | | 0.015 |  |
| Sleep duration |  |  |  | |  |  | | |  |  |
| 7-8 | Reference |  | Reference | |  | Reference | | |  |  |
| < 7 | 1.53 (1.32, 1.78) | < 0.001 | 1.45 (1.24, 1.69) | | < 0.001 | 1.30 (1.11, 1.51) | | | 0.001 |  |
| > 8 | 1.31 (1.10, 1.56) | 0.003 | 1.23 (1.03, 1.46) | | 0.024 | 1.23 (1.03, 1.47) | | | 0.023 |  |
| Model 1 adjusted for nothing. | | | | | | | | |  |  |
| Model 2 adjusted for age, gender, marital status, education, residence region, BMI, smoke, drink, cooking fuel. | | | | | | | | | | |
| Model 3 adjusted for model 2 plus hypertension, diabetes, dyslipidemia, cardiovascular disease and depression. | | | | | | | | | |  |
| S4 Table. Threshold effect analysis for the relationship between sleep duration and CLDs. | | | | | | | |  |  |  |
| CLDs | | | | Adjusted OR (95% CI) | | | P |  |  |  |
| Linear regression model | | | | 0.96 (0.93, 0.99) | | | 0.015 |  |  |  |
| Two-piecewise linear regression model | | | |  | | |  |  |  |  |
| Inflection point | | | | 7, 8 | | |  |  |  |  |
| Sleep duration (h) < 7 | | | | 0.93 (0.88, 0.98) | | | 0.012 |  |  |  |
| Sleep duration (h) 7-8 | | | | 0.85 (0.68, 1.06) | | | 0.150 |  |  |  |
| Sleep duration (h) > 8 | | | | 0.89 (0.79, 1.01) | | | 0.081 |  |  |  |
| Log likelihood ratio test | | | |  | | | < 0.001 |  |  |  |
| CLDs: chronic lung diseases. Adjusted for all covariates. | | | | | | | |  |  |  |

6. **I have read that longer sleep duration (>8 hours) was not significantly associated with CLDs. For this might rather due to low power, I suggest the author to check for insufficient power specifically for this sleeping to group. Recalling my previous comment, categorizing variables, sometimes in non-systematic way may lead to potential under power.**

Thank you very much for your suggestion. The sample size of the group with more than 8 hours of sleep is relatively small, which may have contributed to the lack of positive results. We have revised the results section of the Abstract and removed the negative results for the group with more than 8 hours of sleep. Sleep duration, as a continuous variable, is specially presented in three regression models in Table 2, and sleep duration was associated with CLDs in the full adjusted model.

7. **Overall, I strongly suggest the author to revise the discussion. The discussion should start with the main findings of this study,**

Thank you very much for your suggestions. We have revised the first paragraph of the Discussion section to summarize the main findings and highlights. Marked with green.

8. **and then proceed to discussing each important association in detail. What I missed a lot is that the author did not mention important parts about the literature they cited, for example what data they used, is that cross-sectional, cohort or….design, what years did the study covered, which population group, those are main things to mention,**
9. **otherwise, there is no way for readers to know whether their study is comparative to the literature they cited or not. It is also important to mention whether their findings is in agreement with the literature they cited other than just discussing the literature.**

Thank you very much for your valuable suggestions. We have added relevant content from the literature, including the study design type, study population, data sources, and time frame in the third and fifth paragraphs of the Discussion section. We have also clarified the similarities and differences between our study and other studies int he fourth and sixth paragraphs of the Discussion section, and marked with green.

10. **Finally, I missed the added values of this study. The authors explained quite will in the introduction that there are much study on the same area, however, the added values of this study is not well explained.**

Thank you very much for your suggestions. We have revised the research value section to make it more specific and clear in the first sentence of the seventh paragraph of the Discussion section, and marked it in green.

**Reviewer #2:**

**1. Introduction:**

o Contextual Background: The introduction broadly discusses the prevalence of chronic diseases among older adults in China and the importance of non-smoking risk factors, such as sleep duration. However, it lacks a clear, concise statement of the specific gap in research that this study aims to fill. Clarifying this could help strengthen the rationale for the study.

Thank you for your valuable suggestions. We have revised the description of the research gap to ensure specificity, clarity, and conciseness, thereby strengthening the rationale for this study. The revised content is located in the third paragraph of the Introduction. Mark with yellow.

o Literature Review: The review of existing studies on sleep duration and chronic lung diseases appears somewhat cursory and could benefit from a more detailed discussion. Including a more thorough analysis of previous findings, especially contrasting studies with conflicting results, would provide a stronger foundation for the study's objectives.

Thank you very much for your suggestions. We have revised the literature review, particularly analyzing the possible reasons for the contradictory findings, to lay a solid foundation for the research objectives. The revised content is located in the third paragraph of the Introduction. Mark with yellow.

o Link between Sleep and Lung Health: While the introduction mentions that sleep influences lung health, it could delve deeper into the physiological or biochemical pathways that might mediate this relationship. Providing a brief overview of potential mechanisms could help readers understand why sleep duration might impact chronic lung disease.

Thank you very much for your suggestions. We have added the potential mechanisms by which sleep affects health, particularly the role of inflammatory responses. The revisions have been made in the second paragraph of the Introduction and are highlighted in yellow.

o Epidemiological Data: The introduction cites several statistics regarding chronic diseases and mortality but does not clearly connect these data points specifically to lung health or elaborate on how these figures relate to the study’s focus on sleep duration. A more direct connection between these statistics and the study’s aims would enhance the introduction’s relevance.

Thank you very much for your suggestions. We have revised certain parts to clarify the association between epidemiological data and chronic lung diseases. The revisions are in the first paragraph of the Introduction and are highlighted in yellow.

o Clarity and Focus: The introduction might benefit from tighter editing to improve clarity and focus. Some sentences are overly complex and could be simplified to improve readability and ensure that the study’s aims are immediately clear to the reader.

Thank you very much for your suggestions. To improve clarity and focus, we have removed some less relevant sentences, specifically the first and second sentences of the Introduction.

o Hypothesis or Research Questions: The introduction does not clearly state the research questions or hypotheses. Explicitly stating what the study intends to examine would help to frame the research context and provide a clearer guide for the reader through the rest of the document.

Thank you for your valuable feedback. We have followed your suggestion and added the research hypotheses in the last paragraph of the Introduction, highlighted in green.

**2. Methods:**

Study Design and Population Description:

o The manuscript states that CHARLS is a prospective longitudinal study, yet the study design used for this particular analysis is cross-sectional. This should be clarified to avoid confusion regarding the nature of the data analysis.

Thank you for pointing out this important aspect of the manuscript. We have followed your suggestion and provided a detailed description of the study design type of CHARLS, including the sampling method, in the first paragraph of the Methods section, and marked with green. Additionally, we reiterated our cross-sectional study design in the third paragraph of the Methods section and highlighted these revisions in yellow.

o The inclusion criteria and study population are described, but more detailed justification on the age cut-off and criteria selection could provide better understanding and rationale for their choices.

Thank you for your valuable feedback. We acknowledge the need to provide a more detailed justification for **the age cut-off.** In response, we have elaborated on the rationale for the age cut-off under.

CHARLS targets residents aged 45 and above in China. Since the official retirement age for female workers is 50 years old, and many individuals are eligible for early retirement, setting the age threshold at 45 facilitates the study of the dynamic processes of early retirement and labor force participation. In the overall survey design of the project, including both sampling and questionnaires, CHARLS was rigorously and systematically designed based on international experience while taking into account China's specific context[1]In addition, the WHO defines middle-aged and elderly as follows: Middle-aged Adults: 45-59 years old, Elderly: 60 years old and above.

Selecting middle-aged and elderly populations as the research focus is highly significant, as this period is crucial for studying chronic diseases, mental health issues, and the transition between labor participation and retirement, while also addressing population aging and improving social security policies. Research on this group not only reveals health and social challenges but also provides essential evidence for policymaking and public health interventions to enhance their quality of life.

Thank you for your valuable feedback of **the criteria selection**. We excluded participants with asthma because asthma is a condition related to chronic lung diseases. Additionally, we excluded those with psychological and memory disorders due to the potential for recall bias, which could lead to inaccurate information. Inclusion and exclusion criteria in the revised manuscript in the third paragraph of the Methods section and highlighted these revisions in yellow.

Measurement of Sleep Duration:

o The method of measuring sleep duration combines night sleep and naps without discussing potential biases or the validity of this approach. The impact of napping on overall sleep duration and its potential differential impact on health outcomes needs further discussion or acknowledgment as a limitation.o Only providing average sleep over the past month may not accurately reflect habitual patterns or variability in sleep, which can be crucial in chronic disease frameworks.

Thank you very much for your consideration regarding sleep duration. We referred to relevant literature to define sleep duration[2, 3]. Additionally, some studies have explored the association between nighttime sleep and nap duration with chronic diseases. These studies have pointed out in their limitations that while the two are correlated, it is difficult to fully explain their individual effects on diseases, highlighting the necessity of further investigation into 24-hour sleep patterns[4]. Based on these studies, we used the sum of nighttime sleep and nap duration as the measure of total sleep duration. Of course, We acknowledge that no analyzing the impact of naps on total sleep duration may be a limitation of this study, the revision are marked in yellow in the eighth paragraph of the Discussion section.

In addition, sleep habits and sleep variability may have an impact on the occurrence of diseases. We believe this is a direction for future research and have explained it in the Conclusion section. Mark it with yellow.

Assessment of Chronic Lung Diseases (CLDs):

o CLDs are assessed solely through self-reporting without verification from medical records or diagnostic tests, which could lead to misclassification bias. This limitation is significant and should be addressed more comprehensively in the discussion of the methodological limitations.

Thank you very much for your valuable question. We referred to other literature[5] regarding the definition of CLDs, but through self-reported physician diagnosis it may involve bias and is a limitation of the article. We have further elaborated on this in the eighth paragraph of the Discussion section. Marked with yellow.

Covariate Assessment:

o The selection of covariates is appropriate; however, the manuscript could benefit from a more detailed explanation of why these particular covariates were chosen over others. Additionally, the methods for measuring these variables (e.g., self-report vs. clinical measurement for hypertension and diabetes) need clarification.

Thank you for pointing out this important aspect of the manuscript. We appreciate your suggestion and have now added a more detailed explanation in the Methods section (**Assessment of** **covariates**) regarding the rationale for selecting these covariates. Specifically, these covariates were chosen based on their established relevance in previous studies and their potential influence on the outcomes of interest. Additional references have also been provided to support our selection process. Regarding the measurement methods, we have added the references in the eighth paragraph of the Methods section, highlighted these revisions in yellow.

o The handling of missing data by deletion or dummy variable method may introduce bias, especially if the data are not missing completely at random. More sophisticated methods such as multiple imputation could be considered and discussed.

We greatly appreciate your constructive suggestions. We calculated the number of missing cases and performed multiple imputation. The specific results can be found in the Supporting information (S3 Table, S4 Table). Analysis of the imputed data yielded consistent results, indicating that shorter sleep duration was positivly associated with CLDs, and the turning points at 7 and 8 hours remained evident.

1. Figure shows the missing data pattern of variables

| S3 Table. Logistic regression analysis between sleep duration and chronic lung diseases. | | | | | | |  |
| --- | --- | --- | --- | --- | --- | --- | --- |
| Variables | Model 1 | *P* | Model 2 | *P* | Model 3 | *P* |  |
|  | OR (95% CI) |  | OR (95% CI) |  | OR (95% CI) |  |  |
| Sleep duration | 0.93 (0.90, 0.95) | < 0.001 | 0.93 (0.91, 0.96) | < 0.001 | 0.96 (0.93, 0.99) | 0.015 |  |
| Sleep duration |  |  |  |  |  |  |  |
| 7-8 | Reference |  | Reference |  | Reference |  |  |
| < 7 | 1.53 (1.32, 1.78) | < 0.001 | 1.45 (1.24, 1.69) | < 0.001 | 1.30 (1.11, 1.51) | 0.001 |  |
| > 8 | 1.31 (1.10, 1.56) | 0.003 | 1.23 (1.03, 1.46) | 0.024 | 1.23 (1.03, 1.47) | 0.023 |  |
| Model 1 adjusted for nothing. | | | | | |  |  |
| Model 2 adjusted for age, gender, marital status, education, residence region, BMI, smoke, drink, cooking fuel. | | | | | | | |
| Model 3 adjusted for model 2 plus hypertension, diabetes, dyslipidemia, cardiovascular disease and depression. | | | | | | |  |

| S4 Table. Threshold effect analysis for the relationship between sleep duration and CLDs | | |
| --- | --- | --- |
| CLDs | Adjusted OR (95% CI) | *P* value |
| Linear regression model | 0.96 (0.93, 0.99) | 0.015 |
| Two-piecewise linear regression model |  |  |
| Inflection point | 7, 8 |  |
| Sleep duration (h) < 7 | 0.93 (0.88, 0.98) | 0.012 |
| Sleep duration (h) 7-8 | 0.85 (0.68, 1.06) | 0.150 |
| Sleep duration (h) > 8 | 0.89 (0.79, 1.01) | 0.081 |
| Log likelihood ratio test |  | < 0.001 |
| CLDs: chronic lung diseases. Adjusted for all covariates. | | |

Statistical Analysis:

o The use of logistic regression models is appropriate; however, the decision to use a three-piecewise linear regression model to explore non-linear relationships is briefly mentioned without sufficient justification for this choice. A detailed rationale for selecting this model would strengthen the methodology.

Thank you very much for your detailed observation. Representing it with a three-segment linear regression model is not very accurate. We referred to several articles and modified it to the two-piecewise regression model[6, 7]. Log-likelihood ratio test comparing one-line (non-segmented) model to segmented regression model was used to determine whether threshold exists. This method has been used multiple times in the literature. These revisions were in the tenth paragraph of the Methods section, highlighted in green.

o The manuscript would benefit from additional information on the model fit, diagnostic checks, and whether the assumptions of the used statistical models were met.

Thank you very much for your thorough consideration. For detailed model specifications, please refer to **cld7191_3_tbl.log ( Located at the end of the response)**

Subgroup Analysis and Interaction Tests:

o While subgroup analyses add depth, there is a risk of Type I errors due to multiple comparisons, which does not appear to be addressed. o The manuscript declares a p-value of lss than 0.05 as statistically significant without discussion on the correction for multiple testing, which could be important given the number of analyses conducted.

Thank you very much for your suggestion. We have revised the results section of the Abstract and removed subgroup and interaction analysis results. Since the subgroup analysis serves as a sensitivity analysis, we focused on whether the direction of the effect estimates in the short sleep duration group was consistent with the main analysis results.

o Interaction tests are mentioned, but the criteria for choosing these particular interactions are not discussed. Additionally, the manuscript should clarify how these interactions were tested statistically and interpret their clinical implications.

Thank you very much for your valuable question. Based on relevant literature[5], we selected demographic factors and several variables associated with CLDs to further explore whether these variables influence the relationship between sleep duration and CLDs. These negtive results of the interaction tests showed that these stratifying variables did not modify the association between short sleep duration and CLDs. Our main results are stable. These revisions were in the tenth paragraph of the Methods section, highlighted in yellow.

Additionally, we conducted subgroup analyses and interaction tests for the remaining covariates, and the results were consistent with our main findings. The results are shown in the table below.

Table 33 Subgroup analysis between sleep duration and chronic lung diseases

| Subgroup | Sleep duration (h) | Adjusted OR （95% CI） | P for interaction |
| --- | --- | --- | --- |
| Marital status |  |  | 0.608 |
| Married/living with partner | 7-8 | Reference |  |
|  | < 7 | 1.34 (1.13, 1.59) |  |
|  | > 8 | 1.21 (0.99, 1.47) |  |
| Other | 7-8 | Reference |  |
|  | < 7 | 1.00 (0.66, 1.50) |  |
|  | > 8 | 1.01 (0.62, 1.63) |  |
| Drinking Status |  |  | 0.451 |
| More than once a month | 7-8 | Reference |  |
|  | < 7 | 1.37 (1.00, 1.89) |  |
|  | > 8 | 1.17 (0.81, 1.68) |  |
| Less than once a month | 7-8 | Reference |  |
|  | < 7 | 0.74 (0.41, 1.32) |  |
|  | > 8 | 0.95 (0.50, 1.80) |  |
| Non-drinker | 7-8 | Reference |  |
|  | < 7 | 1.34 (1.10, 1.63) |  |
|  | > 8 | 1.21 (0.97, 1.52) |  |
| Hypertension |  |  | 0.348 |
| Yes | 7-8 | Reference |  |
|  | < 7 | 1.27 (0.99, 1.63) |  |
|  | > 8 | 1.35 (1.02, 1.78) |  |
| No | 7-8 | Reference |  |
|  | < 7 | 1.30 (1.06, 1.60) |  |
|  | > 8 | 1.06 (0.83, 1.36) |  |
| Diabetes |  |  | 0.417 |
| Yes | 7-8 | Reference |  |
|  | < 7 | 1.61 (1.02, 2.53) |  |
|  | > 8 | 1.05 (0.62, 1.80) |  |
| No | 7-8 | Reference |  |
|  | < 7 | 1.26 (1.06, 1.49) |  |
|  | > 8 | 1.21 (0.99, 1.47) |  |
| Dyslipidemia |  |  | 0.107 |
| Yes | 7-8 | Reference |  |
|  | < 7 | 1.17 (0.88, 1.57) |  |
|  | > 8 | 0.90 (0.63, 1.27) |  |
| No | 7-8 | Reference |  |
|  | < 7 | 1.34 (1.11, 1.62) |  |
|  | > 8 | 1.34 (1.08, 1.66) |  |
| Cardiovascular diseases |  |  | 0.745 |
| Yes | 7-8 | Reference |  |
|  | < 7 | 1.07 (0.75, 1.53) |  |
|  | > 8 | 1.10 (0.72, 1.68) |  |
| No | 7-8 | Reference |  |
|  | < 7 | 1.35 (1.13, 1.61) |  |
|  | > 8 | 1.20 (0.98, 1.48) |  |
| Depression |  |  | 0.238 |
| Yes | 7-8 | Reference |  |
|  | < 7 | 1.17 (0.92, 1.48) |  |

Software and Statistical Significance:

o Mentioning the use of STATA and R is helpful, but specific packages or commands used could be included for reproducibility.

Thank you very much for your thorough consideration. Due to the large file size resulting from multiple R packages used, we have only provided the R package for threshold effect analysis (**cld7191_3_tbl.log, Located at the end of the response)**

**3. Results:**

o Cross-sectional Design Limitations: The use of a cross-sectional design limits the ability to infer causality between sleep duration and chronic lung diseases (CLDs). The authors do not sufficiently address this inherent limitation in their methodology section or discuss the potential for reverse causation—whereby individuals with CLDs might experience disturbed sleep due to their condition.

Thank you for your question. The aim of our study was to preliminarily investigate the association between sleep duration and chronic lung diseases. We acknowledge that our cross-sectional design cannot establish causality and the possibility of reverse causation exists, which was described in the eighth paragraph of the Discussion section . However, several retrospective cohort studies have examined that whether sleep duration is associated with the development of chronic diseases[4, 8]. Therefore, our research hypothesis was based on current literature, and the association between sleep duration and chronic diseases have shown inconsistent results across studies.

o Measurement of Sleep Duration: Sleep duration is self-reported, which introduces potential recall bias and misclassification of the exposure variable. The authors do not discuss how this might impact the reliability of their findings or any measures taken to mitigate this bias.

Thank you for your valuable question. We excluded participants who reported psychiatric and memory disorders to enhance the accuracy of questionnaire responses. During the analysis phase, we conducted sensitivity analyses, including subgroup analyses (Table 3) and multivariable regression analysis after multiple imputation (S3 Table, S4 Table), which demonstrated the robustness of our findings. Additionally, we have discussed this limitation in the eighth paragraph of the Discussion section. Marked with yellow.

o Assessment of Chronic Lung Diseases: CLDs are identified solely based on participant self-report without confirmation by medical records or clinical evaluation. This could lead to misdiagnosis or under-reporting of the condition, significantly affecting the study's outcome measurements.

Thank you very much for your valuable question. We referred to other literature[5] regarding the definition of CLDs, but through self-reported physician diagnosis it may involve bias and is a limitation of the article. We have further elaborated on this in the eighth paragraph of the Discussion section. Marked with yellow.

o Confounding Variables: While the authors adjust for a variety of potential confounders in their regression models, there is inadequate discussion regarding the selection and exclusion of these variables. The possibility of residual confounding by unmeasured or inadequately measured variables (e.g., environmental pollutants, occupational exposures) is not addressed.

Thank you for pointing out this important aspect of the manuscript. We appreciate your suggestion and have now added a more detailed explanation in the Methods section ( the first paragraph of Assessment of covariates) regarding the rationale for selecting potential confounding variables, marked with yellow. Specifically, these confounding variables were chosen based on their established relevance in previous studies and their potential influence on the outcomes of interest. Additional references have also been provided to support our selection process.

We appreciate your suggestion for residual confounding. Despite this extensive adjustment, it has still the possibility of residual confounding by unmeasured or inadequately measured variables, which should been acknowledged, and have added a explanation in the eighth paragraph of the Discussion section, marked in yellow.

o Subgroup Analyses: The manuscript reports multiple subgroup analyses without adjusting for multiple comparisons, which increases the risk of Type I errors. The criteria for choosing these particular subgroups for analysis are not well-explained, which might suggest data dredging.

Thank you very much for your valuable question. We have revised the results section of the Abstract and removed subgroup and interaction analysis results. Since the subgroup analysis serves as a sensitivity analysis, we focused on whether the direction of the effect estimates in the short sleep duration group was consistent with the main analysis results. Based on relevant literature[5], we selected demographic factors and several variables associated with CLDs to for stratification. Additionally, we conducted subgroup analyses for the remaining covariates, and the results were consistent with our main findings. The results are shown in the table below.

Table 33 Subgroup analysis between sleep duration and chronic lung diseases

| Subgroup | Sleep duration (h) | Adjusted OR （95% CI） | P for interaction |
| --- | --- | --- | --- |
| Marital status |  |  | 0.608 |
| Married/living with partner | 7-8 | Reference |  |
|  | < 7 | 1.34 (1.13, 1.59) |  |
|  | > 8 | 1.21 (0.99, 1.47) |  |
| Other | 7-8 | Reference |  |
|  | < 7 | 1.00 (0.66, 1.50) |  |
|  | > 8 | 1.01 (0.62, 1.63) |  |
| Drinking Status |  |  | 0.451 |
| More than once a month | 7-8 | Reference |  |
|  | < 7 | 1.37 (1.00, 1.89) |  |
|  | > 8 | 1.17 (0.81, 1.68) |  |
| Less than once a month | 7-8 | Reference |  |
|  | < 7 | 0.74 (0.41, 1.32) |  |
|  | > 8 | 0.95 (0.50, 1.80) |  |
| Non-drinker | 7-8 | Reference |  |
|  | < 7 | 1.34 (1.10, 1.63) |  |
|  | > 8 | 1.21 (0.97, 1.52) |  |
| Hypertension |  |  | 0.348 |
| Yes | 7-8 | Reference |  |
|  | < 7 | 1.27 (0.99, 1.63) |  |
|  | > 8 | 1.35 (1.02, 1.78) |  |
| No | 7-8 | Reference |  |
|  | < 7 | 1.30 (1.06, 1.60) |  |
|  | > 8 | 1.06 (0.83, 1.36) |  |
| Diabetes |  |  | 0.417 |
| Yes | 7-8 | Reference |  |
|  | < 7 | 1.61 (1.02, 2.53) |  |
|  | > 8 | 1.05 (0.62, 1.80) |  |
| No | 7-8 | Reference |  |
|  | < 7 | 1.26 (1.06, 1.49) |  |
|  | > 8 | 1.21 (0.99, 1.47) |  |
| Dyslipidemia |  |  | 0.107 |
| Yes | 7-8 | Reference |  |
|  | < 7 | 1.17 (0.88, 1.57) |  |
|  | > 8 | 0.90 (0.63, 1.27) |  |
| No | 7-8 | Reference |  |
|  | < 7 | 1.34 (1.11, 1.62) |  |
|  | > 8 | 1.34 (1.08, 1.66) |  |
| Cardiovascular diseases |  |  | 0.745 |
| Yes | 7-8 | Reference |  |
|  | < 7 | 1.07 (0.75, 1.53) |  |
|  | > 8 | 1.10 (0.72, 1.68) |  |
| No | 7-8 | Reference |  |
|  | < 7 | 1.35 (1.13, 1.61) |  |
|  | > 8 | 1.20 (0.98, 1.48) |  |
| Depression |  |  | 0.238 |
| Yes | 7-8 | Reference |  |
|  | < 7 | 1.17 (0.92, 1.48) |  |
|  | > 8 | 1.20 (0.89, 1.63) |  |
| No | 7-8 | Reference |  |
|  | < 7 | 1.42 (1.15, 1.76) |  |
|  | > 8 | 1.17 (0.93, 1.48) |  |

o Statistical Methods and Model Fit: The use of a three-piecewise linear regression model to determine inflection points is not sufficiently justified with theoretical or empirical rationale. Furthermore, the authors do not provide diagnostics to evaluate the fit of this model or the logistic regression models used.

Thank you very much for your detailed observation. Representing it with a three-segment linear regression model is not very accurate. We referred to several articles and modified it to the two-piecewise regression model[6, 7]. Log-likelihood ratio test comparing one-line (non-segmented) model to segmented regression model was used to determine whether threshold exists. This method has been used multiple times in the literature.

For detailed model specifications, please refer to file (**cld7191_3_tbl.log, Located at the end of the response)**

o Generalizability: The findings are based on a sample from the China Health and Retirement Longitudinal Study (CHARLS), and the authors claim national representativeness. However, they do not thoroughly discuss the potential limitations in generalizing these findings to other populations or settings outside China.

Thank you for this insightful comment regarding the generalizability of our findings. We have described this limitation in our revised manuscript in the eighth paragraph of the Discussion section, highlighted in green.

o Data Availability: The data are said to be available upon request, which could restrict access for some researchers. The authors do not discuss why the data cannot be deposited in a public repository, which might limit transparency and reproducibility of the research.

Thank you for raising this important point about data accessibility. We have revised our data availability statement in our revised manuscript in Data availability section, highlighted in yellow, as follows: The raw data for this study is available from: http://charls.pku.edu.cn/en. The dataset generated from this study can be obtained from the supporting information.

o Ethical Considerations: The study involves human participants, and while it mentions ethical approval, there is little discussion on the consent process, particularly how informed consent was obtained from participants or if any special considerations were made for vulnerable populations like the elderly.

Thank you for your valuable comment regarding ethical considerations. Our study is a secondary analysis of the data, and CHARLS was approved by the Ethics Review Committee of Peking University. The ethical approval number is IRB00001052–11015. Written informed consent was obtained from all participants.

o Potential Bias in Authorship: The study lists only one author, which could raise concerns about the breadth of expertise and potential biases in conducting the research and interpreting the findings. Collaborative research often benefits from diverse perspectives, particularly in multidisciplinary fields like epidemiology and public health.

Thank you for your thoughtful comment regarding authorship and potential bias. To address your concern, we have incorporated a clinical specialist who reviewed our study design and hypotheses, and contributed significantly to revising the Introduction and Discussion sections. Furthermore, we received methodological help from a statistical expert who provided valuable suggestions for our analysis.

**4. Discussion:**

o Causal Inferences: The authors discuss the results as if they imply causality between sleep duration and chronic lung diseases (CLDs). Given the cross-sectional nature of the study, such inferences are inappropriate as they cannot establish causality due to the simultaneous measurement of exposure and outcome. This limitation should be acknowledged more robustly, with a clearer statement that the findings can only suggest association, not causation.

Thank you for your valuable comments. We fully agree with your point regarding causal inferences, and have made the following revisions.

1.Explicitly acknowledged the limitation of cross-sectional design in inferring causality in the eighth paragraph of the Discussion section, highlighted in yellow.

2.Revised terminology, replacing words suggesting causality (e.g., "cause," "effect") with terms like "association" and "relationship"

3.Emphasized the need for prospective studies to verify causal relationships in the Conclusion section. Marked with yellow.

o Comparison with Other Studies: While the discussion mentions various studies that either support or contradict the findings, there is a lack of critical analysis regarding why these differences might exist. The authors could enhance the discussion by exploring potential reasons for discrepancies, such as differences in study design, population characteristics, or methods of measuring sleep duration and lung health.

Thank you very much for your suggestion. As you mentioned, we have thoroughly discussed the reasons for the differences in findings across studies in the sixth paragraph of the Discussion section, marked in green.

o Generalizability: The authors claim that their findings are representative of the broader Chinese population aged 45 and over. However, they do not adequately address the potential for selection bias or the impact of regional variations within China that might affect the generalizability of the results. A more detailed discussion on the representativeness of the sample and its implications for applying these findings to different subgroups or geographic areas would be beneficial.

Thank you very much for your suggestion. CHARLS uses a multistage stratified probability proportion to size sampling method to ensure a well-represented sample. Our study focused on middle-aged and elderly Chinese people, the conclusions are also limited to this specific population, We acknowledge that the generalizability of our findings is limited in the the eighth paragraph of the Discussion section, marked in green.

o Mechanistic Insights: The discussion provides a general overview of the physiological mechanisms by which sleep might influence lung health, such as through inflammation and immune function. However, these explanations are somewhat superficial and generic. The section would benefit from a more detailed, specific discussion of the mechanisms potentially linking sleep duration with the specific types of chronic lung disease observed, possibly incorporating recent research or theoretical models.

Thank you very much for your suggestions. We have revised our discussion of the mechanisms and added relevant references to provide a more in-depth explanation of how inflammation, particularly inflammatory factors, may mediate the association between sleep and chronic lung disease. These changes are highlighted in green in the fourth paragraph of the Discussion section

o Methodological Limitations: The discussion touches on the limitations of the study but does not critically engage with how these might have influenced the results. For instance, the reliance on self-reported sleep and disease measures could introduce significant bias, which is not thoroughly analyzed. Discussing the potential impact of these biases and how they might be mitigated in future research would strengthen the section.

Thank you very much for your detailed and valuable suggestions. Based on your advice, we have provided a detailed explanation in the limitations section of the discussion, specifically in the eighth paragraph of the Discussion section, marked in yellow/green.

o Implications for Public Health: While the discussion suggests potential public health interventions, it does so without a clear strategy for implementation or consideration of the challenges that might be faced in promoting sleep health among middle-aged and older adults. A more nuanced discussion on the practical steps, potential barriers, and necessary collaborations for effective intervention would provide valuable insights for readers.

Thank you for your insightful comments regarding public health implementation strategies. In response to your concerns about: a more nuanced discussion on the practical steps, potential barriers, and necessary collaborations for effective intervention, we have revised in the seventh paragraph of the Discussion section, marked in green/yellow.

o Future Research Directions: The section could be improved by offering more concrete suggestions for future research, such as longitudinal studies or clinical trials that could validate the findings and provide stronger evidence for causality. Additionally, exploring other relevant factors such as sleep quality and its interaction with sleep duration could be suggested.

Thank you very much for your suggestions. We have added the corresponding content and marked it in yellow in the Conclusion section.

5. Limitations:

o Self-Reported Measures: The study heavily relies on self-reported data for both sleep duration and the diagnosis of chronic lung diseases. Self-reported data can be subject to recall bias and misclassification, which might affect the study's accuracy and reliability. Discussing the potential impact of these biases and how they might affect the findings would provide a more comprehensive view of the study's limitations.

o Cross-Sectional Design: While you briefly mention the inability to establish causality due to the cross-sectional design, this limitation deserves more emphasis. It's crucial to discuss how this design restricts the ability to discern whether reduced sleep actually leads to increased incidence of chronic lung diseases or if perhaps individuals with such diseases might experience sleep disturbances due to their condition.

o Lack of Polysomnography Data: The study uses a simplified measure of sleep duration without validation through more objective measures such as polysomnography, which can provide detailed insights into sleep quality and architecture. Mentioning the lack of such data as a limitation would be beneficial, as it could affect the interpretation of the relationship between sleep quality and lung health.

o Generalizability: The findings are based on a specific demographic (Chinese middle-aged and older adults), which may not be generalizable to other populations or age groups. Expanding on the limitations in terms of ethnic and demographic diversity could highlight the need for caution when extrapolating these results to other populations.

o Confounding Variables: While the study adjusts for numerous potential confounders, there might be unmeasured or residual confounding factors such as environmental or occupational exposures that can influence both sleep and lung health. More discussion on the possibility of such unmeasured confounders would strengthen the limitations section.

o Temporal Fluctuations in Sleep Patterns: The study does not account for changes in sleep patterns over time, which can be particularly relevant in an aging population. The inability to track changes in sleep duration over time and its potential impact on the development of chronic conditions is a notable limitation.

o Details on Data Collection Methods: More detailed criticism could be levied at the methods used for data collection, particularly if the sleep data collection did not differentiate between weekdays and weekends or consider variability in sleep patterns, which could influence the overall sleep duration reported.

Thank you very much for your detailed and valuable suggestions about the Limitation section. Based on your advice, we have provided a detailed explanation in the the eighth paragraph of the Discussion section, marked in yellow/green.

6. Ethical Considerations:

o The ethical approval and consent procedures are adequately described. However, a statement about how the data were anonymized or any other data security measures taken during the analysis would enhance the ethical reporting.

Thank you very much for your valuable suggestions. This study is based on publicly available de-identified data.

7. Conclusion:

o The conclusion succinctly summarizes the findings, but it could better highlight the practical implications of the research, suggesting specific public health interventions or policy changes that could be informed by the study results.

Thank you very much for your suggestions. In the Conclusion section, we have added public health intervention strategies based on our study results, marked in yellow.

**References:**

[1].	Yao Hui Zhao, Y.W.X.C., China Health and Retirement Report. 2019. 206.

[2].	Chen, X., et al., Relationship between sleep duration and sociodemographic characteristics, mental health and chronic diseases in individuals aged from 18 to 85 years old in Guangdong province in China: a population-based cross-sectional study. BMC Psychiatry, 2020. 20(1): p. 455.

[3].	Wang, S., et al., Sleep duration and its association with demographics, lifestyle factors, poor mental health and chronic diseases in older Chinese adults. Psychiatry Research, 2017. 257: p. 212-218.

[4].	Wang, Y., et al., Effects and differences of sleep duration on the risk of new-onset chronic disease conditions in middle-aged and elderly populations. European Journal of Internal Medicine, 2023. 107: p. 73-80.

[5].	Yang, K. and R. Chen, Association between cooking fuel exposure and respiratory health: Longitudinal evidence from the China Health and Retirement Longitudinal Study (CHARLS). Ecotoxicology and Environmental Safety, 2024. 275: p. 116247.

[6].	Li, W., et al., A novel U-shaped relationship between BMI and risk of generalized aggressive periodontitis in Chinese: A cross-sectional study. J Periodontol, 2019. 90(1): p. 82-89.

[7].	Chen, C. and J.L. Dai, Triglyceride to high-density lipoprotein cholesterol (HDL-C) ratio and arterial stiffness in Japanese population: a secondary analysis based on a cross-sectional study. Lipids Health Dis, 2018. 17(1): p. 130.

[8].	Yang, M.S., et al., A prospective study on association between sleep duration and the risk of chronic obstructive pulmonary disease in adults in Suzhou. Zhonghua Liu Xing Bing Xue Za Zhi, 2024. 45(3): p. 331-338.

**cld7191_3_tbl.log**

R version 4.2.0 (2022-04-22 ucrt) -- "Vigorous Calisthenics"

Copyright (C) 2022 The R Foundation for Statistical Computing

Platform: x86_64-w64-mingw32/x64 (64-bit)

R is free software and comes with ABSOLUTELY NO WARRANTY.

You are welcome to redistribute it under certain conditions.

Type 'license()' or 'licence()' for distribution details.

R is a collaborative project with many contributors.

Type 'contributors()' for more information and

'citation()' on how to cite R or R packages in publications.

Type 'demo()' for some demos, 'help()' for on-line help, or

'help.start()' for an HTML browser interface to help.

Type 'q()' to quit R.

> ## Created by EmpowerStats @ Fri, 17 Jan 25 09:02:51 +0100##

> #******************** Regarding ALL Following R Code	********************

> #*** COPYRIGHT (c) 2010, 2021 X&Y Solutions, ALL RIGHT RESERVED ***********

> #******************* www.EmpowerStats.com *********************************

> #**************************************************************************

> Sys.setlocale(category = 'LC_ALL', locale = 'English_United States.1252');

[1] "LC_COLLATE=English_United States.1252;LC_CTYPE=English_United States.1252;LC_MONETARY=English_United States.1252;LC_NUMERIC=C;LC_TIME=English_United States.1252"

Warning message:

In Sys.setlocale(category = "LC_ALL", locale = "English_United States.1252") :

using locale code page other than 65001 ("UTF-8") may cause problems

> .libPaths(file.path(R.home(),'library'));

> library(doBy);

Warning message:

package 'doBy' was built under R version 4.2.3

> options(timeout=600);

> library(plotrix);

Warning message:

package 'plotrix' was built under R version 4.2.3

> library(stringi);

Warning message:

package 'stringi' was built under R version 4.2.3

> library(stringr);

Warning message:

package 'stringr' was built under R version 4.2.3

> library(survival);

> library(rms);

Loading required package: Hmisc

Attaching package: 'Hmisc'

The following objects are masked from 'package:base':

format.pval, units

Warning messages:

1: package 'rms' was built under R version 4.2.3

2: package 'Hmisc' was built under R version 4.2.3

> library(nnet);

> library(car);

Loading required package: carData

Attaching package: 'car'

The following objects are masked from 'package:rms':

Predict, vif

Warning messages:

1: package 'car' was built under R version 4.2.3

2: package 'carData' was built under R version 4.2.3

> library(mgcv);

Loading required package: nlme

This is mgcv 1.8-40. For overview type 'help("mgcv-package")'.

Attaching package: 'mgcv'

The following object is masked from 'package:nnet':

multinom

> pdfwd<-6; pdfht<-6;

> load('D:/EmpowerXYS/A123/Analysis/cld7191/cld7191.Rdata');

> if (length(which(ls()=='EmpowerStatsR'))==0) EmpowerStatsR<-get(ls()[1]);

> names(EmpowerStatsR)<-toupper(names(EmpowerStatsR));

> originalVNAME<-names(EmpowerStatsR);

> ofname<-'cld7191_3_tbl';

> recodevar <- function (var,oldcode,newcode) {

+	tmp.v <- var

+	nc.tmp <- length(oldcode)

+	str <- 0;

+	if (!is.numeric(oldcode)) {

+	if (sum(grepl('[|(|)|]|^|*|+|$|.|\\|/', oldcode))==0) {

+	if (sum(duplicated(substr(oldcode,1,min(nchar(oldcode)))))==0) {oldcode<-paste("^",oldcode,sep=""); str <- 1;}

+	}

+	}

+	for (i in (1:nc.tmp)) {

+	if (!is.na(oldcode[i]) & oldcode[i] != "NA") {

+	if (str == 1) {tmp.v[str_detect(var, str_escape('(0,19]'))] <- newcode[i];

+	} else {tmp.v[var == oldcode[i]]<-newcode[i]; }

+	} else if (!is.na(newcode[i])) {tmp.v[is.na(var)] <- newcode[i] }

+	}

+	if (is.factor(tmp.v)) {tmp.v1<-as.numeric(as.character(tmp.v))} else {tmp.v1<-as.numeric(tmp.v)}

+	rm(tmp.v); return(tmp.v1)

+ }

> attach(EmpowerStatsR);

> sink(paste(ofname,'_datastep.lst',sep=''));

> print('Creating new variable: AGE.C');

> AGE.C<- 0+(AGE>=60);

> AGE.C[is.na(AGE)]<- NA;

> summary(AGE.C);

> EmpowerStatsR<-cbind(EmpowerStatsR,AGE.C);

> print('Creating new variable: BMI.C');

> BMI.C<- 0+(BMI>=24);

> BMI.C[is.na(BMI)]<- NA;

> summary(BMI.C);

> EmpowerStatsR<-cbind(EmpowerStatsR,BMI.C);

> print('Creating new variable: BMI.C.RCD');

> BMI.C.RCD<- recodevar(BMI.C,c(0,1,NA),c(0,1,2));

> summary(BMI.C.RCD);

> EmpowerStatsR<-cbind(EmpowerStatsR,BMI.C.RCD);

> rm(AGE.C,BMI.C,BMI.C.RCD);

> sink();

> vname<-c(NA,'GENDER','GENDER.0','GENDER.1','MARRY','MARRY.0','MARRY.1','RURAL','RURAL.0','RURAL.1','LUNGE','LUNGE.0','LUNGE.1','BMI','AGE','EDU','EDU.1','EDU.2','EDU.3','EDU.4','DIABE1','DIABE1.0','DIABE1.1','DRINK','DRINK.1','DRINK.2','DRINK.3','COOKINGF','COOKINGF.0','COOKINGF.1','DEPRESSION','DEPRESSION.0','DEPRESSION.1','HYPERTION','HYPERTION.0','HYPERTION.1','DYSLIPE1','DYSLIPE1.0','DYSLIPE1.1','SMOKE','SMOKE.0','SMOKE.1','SMOKE.2','SLEEP1','CVD','CVD.0','CVD.1','AGE.C','AGE.C.0','AGE.C.1','BMI.C','BMI.C.0','BMI.C.1','BMI.C.RCD','BMI.C.RCD.0','BMI.C.RCD.1','BMI.C.RCD.2')[-1];

> vlabel<-c(NA,'GENDER',' 0',' 1','MARRY',' 0',' 1','RURAL',' 0',' 1','LUNGE',' 0',' 1','BMI','AGE','EDU',' 1',' 2',' 3',' 4','DIABE1',' 0',' 1','DRINK',' 1',' 2',' 3','COOKINGF',' 0',' 1','DEPRESSION',' 0',' 1','HYPERTION',' 0',' 1','DYSLIPE1',' 0',' 1','SMOKE',' 0',' 1',' 2','SLEEP1','CVD',' 0',' 1','AGE categorical',' <60',' >=60','BMI categorical',' <24',' >=24','BMI categorical recoded',' 0',' 1',' 2')[-1];

> varused4this <- c('GENDER','MARRY','RURAL','LUNGE','BMI','AGE','EDU','DIABE1','DRINK','COOKINGF','DEPRESSION','HYPERTION','DYSLIPE1','SMOKE','SLEEP1','CVD','AGE.C','BMI.C','BMI.C.RCD');

> pkgs<-c('MASS','gdata','geepack','mgcv');

> for (g in pkgs) {

+ if (!(g %in% rownames(installed.packages()))) install.packages(g,repos='https://cloud.r-project.org');

+ }

> library(MASS);

> library(gdata);

Attaching package: 'gdata'

The following object is masked from 'package:stats':

nobs

The following object is masked from 'package:utils':

object.size

The following object is masked from 'package:base':

startsWith

Warning message:

package 'gdata' was built under R version 4.2.3

> library(geepack);

Warning message:

package 'geepack' was built under R version 4.2.3

> library(mgcv);

> WD <- EmpowerStatsR; rm(EmpowerStatsR); gc();

used (Mb) gc trigger (Mb) max used (Mb)

Ncells 2331616 124.6	4853762 259.3 2767170 147.8

Vcells 4153602 31.7	8388608 64.0 5357273 40.9

> title<-'阈值效应分析';

> wd.subset='';

> weights.var<- NA;

> yvname<-c('LUNGE');

> ydist<-c('binomial');

> ylink<-c('logit');

> ylv<-c(2);

> par1<-'7 8';

> avname<-c('AGE.C','GENDER','MARRY','RURAL','EDU','SMOKE','DRINK','COOKINGF','BMI.C.RCD','HYPERTION','DYSLIPE1','DIABE1','CVD','DEPRESSION');

> saf<-c(0,0,0,0,0,0,0,0,0,0,0,0,0,0);

> alv<-c(2,2,2,2,4,3,3,2,3,2,2,2,2,2);

> bvar<- NA;

> xvname<-c('SLEEP1');

> xlv<-c(0);

> chk<- 0;

> cox<- 0;

> timevar<- NA;

> vname.start<- NA;

> subjvname<- NA;

> gee.TYPE<-c('independence', 'unstructured', 'exchangeable', 'AR1')[0];

> prn<-1;

> dec<-2;

> ##R package## MASS gdata geepack mgcv ##R package##;

> pvformat<-function(p,dec) {

+	pp <- sprintf(paste("%.",dec,"f",sep=""),as.numeric(p))

+	if (is.matrix(p)) {pp<-matrix(pp, nrow=nrow(p)); colnames(pp)<-colnames(p);rownames(pp)<-rownames(p);}

+	lw <- paste("<",substr("0.00000000000",1,dec+1),"1",sep="");

+	pp[as.numeric(p)<(1/10^dec)]<-lw

+	return(pp)

+ }

> numfmt<-function(p,dec) {

+	if (is.list(p)) p<-as.matrix(p)

+	pp <- sprintf(paste("%.",dec,"f",sep=""),as.numeric(p))

+	if (is.matrix(p)) {pp<-matrix(pp, nrow=nrow(p));colnames(pp)<-colnames(p);rownames(pp)<-rownames(p);}

+	pp[as.numeric(p)>10000000]<- "inf."

+	pp[is.na(p) | gsub(" ","",p)==""]<- ""

+	pp[p=="-Inf"]<-"-Inf"

+	pp[p=="Inf"]<-"Inf"

+	return(pp)

+ }

> mat2htmltable<-function(mat) {

+	t1<- apply(mat,1,function(z) paste(z,collapse="</td><td>"))

+	t2<- paste("<tr><td>",t1,"</td></tr>")

+	return(paste(t2,collapse=" "))

+ }

> setgam<-function(fml,yi,wdtmp) {

+	if (ydist[yi]=="") ydist[yi]<-"gaussian"

+	if (ydist[yi]=="exact") ydist[yi]<-"binomial"

+	if (ydist[yi]=="breslow") ydist[yi]<-"binomial"

+	if (ydist[yi]=="gaussian") mdl<-try(gam(formula(fml),weights=wdtmp$weights,data=wdtmp, family=gaussian(link="identity")))

+	if (ydist[yi]=="binomial") mdl<-try(gam(formula(fml),weights=wdtmp$weights,data=wdtmp, family=binomial(link="logit")))

+	if (ydist[yi]=="poisson") mdl<-try(gam(formula(fml),weights=wdtmp$weights,data=wdtmp, family=poisson(link="log")))

+	if (ydist[yi]=="gamma") mdl<-try(gam(formula(fml),weights=wdtmp$weights,data=wdtmp, family=Gamma(link="inverse")))

+	if (ydist[yi]=="negbin") mdl<-try(gam(formula(fml),weights=wdtmp$weights,data=wdtmp, family=negbin(c(1,10), link="log")))

+	return(mdl)

+ }

> setgee<-function(fml,yi, wdtmp) {

+	if (ydist[yi]=="") ydist[yi]<-"gaussian"

+	if (ydist[yi]=="exact") ydist[yi]<-"binomial"

+	if (ydist[yi]=="breslow") ydist[yi]<-"binomial"

+	if (ydist[yi]=="gaussian") md<-try(geeglm(formula(fml),id=wdtmp[,subjvname],corstr=gee.TYPE,family="gaussian",weights=wdtmp$weights,data=wdtmp))

+	if (ydist[yi]=="binomial") md<-try(geeglm(formula(fml),id=wdtmp[,subjvname],corstr=gee.TYPE,family="binomial",weights=wdtmp$weights,data=wdtmp))

+	if (ydist[yi]=="poisson") md<-try(geeglm(formula(fml),id=wdtmp[,subjvname],corstr=gee.TYPE,family="poisson",weights=wdtmp$weights,data=wdtmp))

+	if (ydist[yi]=="gamma") md<-try(geeglm(formula(fml),id=wdtmp[,subjvname],corstr=gee.TYPE,family="Gamma",weights=wdtmp$weights,data=wdtmp))

+	if (ydist[yi]=="negbin") md<-try(geeglm.nb(formula(fml),id=wdtmp[,subjvname],corstr=gee.TYPE,weights=wdtmp$weights,data=wdtmp))

+	return(md)

+ }

> setglm<-function(fml,yi, wdtmp) {

+	if (ydist[yi]=="") ydist[yi]<-"gaussian"

+	if (ydist[yi]=="exact") ydist[yi]<-"binomial"

+	if (ydist[yi]=="breslow") ydist[yi]<-"binomial"

+	if (ydist[yi]=="gaussian") md<-try(glm(formula(fml),family="gaussian",weights=wdtmp$weights,data=wdtmp))

+	if (ydist[yi]=="binomial") md<-try(glm(formula(fml),family="binomial",weights=wdtmp$weights,data=wdtmp))

+	if (ydist[yi]=="poisson") md<-try(glm(formula(fml),family="poisson",weights=wdtmp$weights,data=wdtmp))

+	if (ydist[yi]=="gamma") md<-try(glm(formula(fml),family="Gamma",weights=wdtmp$weights,data=wdtmp))

+	if (ydist[yi]=="negbin") md<-try(glm.nb(formula(fml),weights=wdtmp$weights,data=wdtmp))

+	return(md)

+ }

> mdl2oo<-function(mdl, xxname, opt) {

+	if (is.na(mdl[[1]][1])) return(rep(" ",times=length(xxname)))

+	if (substr(mdl[[1]][1],1,5)=="Error") return(rep(" ",times=length(xxname)))

+	decp<-dec+2; if (decp>4) decp<-4

+	gs<-summary(mdl); print(mdl$formula); print(gs)

+	if (opt=="gam") {gsparm <- gs$p.table; } else {gsparm <- gs$coefficients;}

+	gsp<-gsparm[match(xxname,rownames(gsparm)),]

+	if (length(xxname)==1) {beta<-gsp[1]; se<-gsp[2]; pv<-gsp[4];

+	} else {beta<-gsp[,1]; se<-gsp[,2]; pv<-gsp[,4]; }

+	ci1<- beta-1.96*se; ci2<- beta+1.96*se

+	pvx<-substr(rep("****",length(pv)),1,(pv<=0.05)+(pv<=0.01)+(pv<=0.001))

+	if (colprn==3) {pvv<-pvx;} else {pvv<-pvformat(pv,decp);}

+	if ((colprn!=2) & (gs$family[[2]]=="log" | gs$family[[2]]=="logit")) {

+	o1<-paste(numfmt(exp(beta),dec)," (",numfmt(exp(ci1),dec),", ",numfmt(exp(ci2),dec),")",sep="")

+	} else {

+	if (colprn<3) {o1<-paste(numfmt(beta,dec), " (",numfmt(ci1,dec),", ",numfmt(ci2,dec),")",sep="")

+	} else {o1<-paste(numfmt(beta,dec), "+",numfmt(se,dec),sep="");}

+	}

+	o1<-paste(o1,pvv); o1[is.na(beta)]<-NA

+	return(o1)

+ }

> removeNA<-function(i,j,wdf) {

+ vvv<-c(yvname[i],xvname[j],avname,subjvname,bvar,vname.start,timevar);

+ vvv<-vvv[!is.na(vvv)]; vvv<-vvv[vvv>" "]

+ tmp<-is.na(wdf[,vvv]);

+ return(wdf[apply(tmp,1,sum)==0,])

+ }

> get.tpval<-function(i,j,g,opt,wdtmp, tppmin=NA, tppmax=NA) {

+	if (sum(is.na(wdtmp))>0) {

+	if (is.na(g)) {wdtmp<-removeNA(i,j,WD);

+	} else if (g<nblv) {wdtmp0<-WD[WD[,bvar]==blv[g],]; wdtmp<-removeNA(i,j,wdtmp0);

+	} else {wdtmp<-removeNA(i,j,WD); }

+	}

+	if (is.na(g)) {fmladj1<-fmladj;

+	} else if (g<nblv) {fmladj1<-fmladj;

+	} else {fmladj1<-paste(fmladj,"+factor(",bvar,")",sep="");}

+	xTMP <- wdtmp[,xvname[j]]

+	tmp.ss<-seq(0.05,0.95,0.05)

+	tp<-quantile(xTMP,probs=tmp.ss,na.rm=TRUE)

+	tmp.llk<-rep(NA,length(tmp.ss))

+	fml<-paste(yvname[i],"~",xvname[j],"+tmp.X",fmladj1)

+	if (!is.na(tppmin) & !is.na(tppmax)) {

+	tp2.min = tppmin; tp2.max = tppmax;

+	} else {

+	for (k in (1:length(tmp.ss))) {

+	tmp.X<-(xTMP > tp[k])*(xTMP-tp[k]); wdtmp1<-cbind(wdtmp,tmp.X)

+	if (opt=="glm" | opt=="gee") tmp.mdl<-setglm(fml, i, wdtmp1);

+	if (opt=="gam") tmp.mdl<-setgam(fml, i, wdtmp1);

+	tmp.llk[k]<-logLik(tmp.mdl)

+	rm(wdtmp1, tmp.X)

+	}

+	tp1<-tmp.ss[which.max(tmp.llk)]

+	tp2.min = tp1 - 0.04

+	tp2.max = tp1 + 0.04

+	if (tp2.min<0.05) {tp2.min=0.05}

+	if (tp2.max>0.95) {tp2.max=0.95}

+	}

+	tp.pctlrange<-quantile(xTMP,probs=c(tp2.min,tp2.max),na.rm=TRUE)

+	tp.range<-unique(xTMP[xTMP>tp.pctlrange[1] & xTMP<tp.pctlrange[2]])

+	while (length(tp.range)>5) {

+	tmp.pct3<-quantile(tp.range,probs=c(0,0.25,0.5,0.75,1),type=3)

+	tmp.llk3<-rep(NA,3)

+	for (k in (2:4)) {

+	tmp.X<-(xTMP>tmp.pct3[k])*(xTMP-tmp.pct3[k]); wdtmp1<-cbind(wdtmp,tmp.X)

+	if (opt=="glm" | opt=="gee") tmp.mdl<-setglm(fml, i, wdtmp1);

+	if (opt=="gam") tmp.mdl<-setgam(fml, i, wdtmp1);

+	tmp.llk3[k-1]<-logLik(tmp.mdl)

+	rm(wdtmp1, tmp.X)

+	}

+	tmp.min3<-which.max(tmp.llk3)

+	tp.range<-tp.range[tp.range>=tmp.pct3[tmp.min3] & tp.range<=tmp.pct3[tmp.min3+2]]

+	}

+	if (length(tp.range)>0) {

+	if (length(tp.range)==1) {tp.val=tp.range[1];} else {

+	tmp.llk<-rep(NA,length(tp.range))

+	for (k in (1:length(tp.range))) {

+	tmp.X<-(xTMP>tp.range[k])*(xTMP-tp.range[k]); wdtmp1<-cbind(wdtmp,tmp.X)

+	if (opt=="glm" | opt=="gee") tmp.mdl<-setglm(fml, i, wdtmp1);

+	if (opt=="gam") tmp.mdl<-setgam(fml, i, wdtmp1);

+	tmp.llk[k]<-logLik(tmp.mdl)

+	rm(wdtmp1, tmp.X)

+	}

+	tp.val<-tp.range[which.max(tmp.llk)]

+	}

+	} else { tp.val<-tp.pctlrange[1];}

+	return(round(tp.val,dec));

+ }

> get2lines<-function(i,j,g,tp.value,opt) {

+	if (is.na(g)) {fmladj1<-fmladj;wdtmp<-removeNA(i,j,WD);

+	} else if (g<nblv) {fmladj1<-fmladj;wdtmp0<-WD[WD[,bvar]==blv[g],];wdtmp<-removeNA(i,j,wdtmp0);

+	} else {fmladj1<-paste(fmladj,"+factor(",bvar,")",sep="");wdtmp<-removeNA(i,j,WD);}

+	xTMP<-wdtmp[,xvname[j]]

+	tmp.X1<-(xTMP<=tp.value)*(xTMP-tp.value)

+	tmp.X2<-(xTMP> tp.value)*(xTMP-tp.value)

+	wdtmp1<-cbind(wdtmp,xTMP,tmp.X1,tmp.X2)

+	fml0<-paste(yvname[i],"~xTMP+tmp.X2",fmladj1)

+	fml1<-paste(yvname[i],"~tmp.X1+tmp.X2",fmladj1)

+	fml2<-paste(yvname[i],"~xTMP",fmladj1)

+	fmlp<-paste(yvname[i],"~xTMP+tmp.X2")

+	tmpn<-nrow(wdtmp)

+	print(paste("tp.value ==", tp.value))

+	if (opt=="glm") {

+	tmp.mdl0<-setglm(fml0,i,wdtmp1); tmp.mdl1<-setglm(fml1,i,wdtmp1)

+	tmp.mdl2<-setglm(fml2,i,wdtmp1); tmp.mdlp<-setglm(fmlp,i,wdtmp1)

+	}

+	if (opt=="gam") {

+	tmp.mdl0<-setgam(fml0,i,wdtmp1); tmp.mdl1<-setgam(fml1,i,wdtmp1)

+	tmp.mdl2<-setgam(fml2,i,wdtmp1); tmp.mdlp<-setgam(fmlp,i,wdtmp1)

+	}

+	if (opt=="gee") {

+	tmp.mdl0<-setgee(fml0,i,wdtmp1); tmp.mdl1<-setgee(fml1,i,wdtmp1)

+	tmp.mdl2<-setgee(fml2,i,wdtmp1); tmp.mdlp<-setglm(fmlp,i,wdtmp1)

+	}

+	pd<-predict(tmp.mdlp,data.frame(xTMP=tp.value,tmp.X2=0), se.fit=TRUE)

+	prd<-paste(numfmt(pd$fit,dec)," (",numfmt(pd$fit-1.96*pd$se.fit,dec),", ", numfmt(pd$fit+1.96*pd$se.fit, dec),")",sep="")

+	m2<-mdl2oo(tmp.mdl2,"xTMP",opt)

+	m1<-mdl2oo(tmp.mdl1,c("tmp.X1","tmp.X2"),opt)

+	m0<-mdl2oo(tmp.mdl0,"tmp.X2",opt)

+	if (opt=="gee") {

+	plrt<-try(anova(tmp.mdl0,tmp.mdl2)$"P(>|Chi|)",TRUE)

+	plrt<-ifelse((plrt<="9" && plrt>="0"), pvformat(plrt,3),"-")

+	} else {plrt<- pvformat(1-pchisq(2*(logLik(tmp.mdl0)[1]-logLik(tmp.mdl2)[1]),1),3);}

+	plrti<-""; plrtx<-""

+	if (!is.na(g) & g==nblv) {

+	fmli<-paste(yvname[i],"~xTMP+xTMP*factor(",bvar,")",fmladj)

+	fmlx<-paste(yvname[i],"~tmp.X1+factor(",bvar,")*tmp.X1+tmp.X2+factor(",bvar,")*tmp.X2",fmladj)

+	if (opt=="gee") {

+	tmp.mdli<-setgee(fmli,i,wdtmp1)

+	tmp.mdlx<-setgee(fmlx,i,wdtmp1)

+	} else {

+	if (opt=="glm") {tmp.mdli<-setglm(fmli,i,wdtmp1);tmp.mdlx<-setglm(fmlx,i,wdtmp1);}

+	if (opt=="gam") {tmp.mdli<-setgam(fmli,i,wdtmp1);tmp.mdlx<-setgam(fmlx,i,wdtmp1);}

+	plrti<- paste("P-interaction:", pvformat(1-pchisq(2*(logLik(tmp.mdli)[1]-logLik(tmp.mdl2)[1]),nblv-2),3));

+	plrtx<- paste("P-interaction:", pvformat(1-pchisq(2*(logLik(tmp.mdlx)[1]-logLik(tmp.mdl1)[1]),2*(nblv-2)),3));

+	}

+	print(summary(tmp.mdli));

+	print("Interaction model === >")

+	print(paste("tp.value ==", tp.value))

+	print(summary(tmp.mdlx))

+	}

+	oo<-list(c(plrti,m2,plrtx,tp.value,m1,m0,prd,plrt),tmpn)

+	return(oo)

+ }

> get3lines<-function(i,j,g,tp.value,opt) {

+	if (is.na(g)) {fmladj1<-fmladj;wdtmp<-removeNA(i,j,WD);

+	} else if (g<nblv) {fmladj1<-fmladj;wdtmp0<-WD[WD[,bvar]==blv[g],];wdtmp<-removeNA(i,j,wdtmp0);

+	} else {fmladj1<-paste(fmladj,"+factor(",bvar,")",sep="");wdtmp<-removeNA(i,j,WD);}

+	xTMP<-wdtmp[,xvname[j]]; tp1<-tp.value[1]; tp2<-tp.value[2]

+	tmp.X1<- (xTMP< tp1)*(xTMP-tp1)

+	tmp.X2<-((xTMP>=tp1) & (xTMP<=tp2))*(xTMP-tp1)

+	tmp.X3<- (xTMP> tp2)*(xTMP-tp2)

+	tmp.B1<- (xTMP>tp2)

+	wdtmp1<-cbind(wdtmp,xTMP,tmp.X1,tmp.X2,tmp.X3,tmp.B1)

+	fml0<-paste(yvname[i],"~xTMP+tmp.X1+tmp.X3+tmp.B1",fmladj1)

+	fml1<-paste(yvname[i],"~tmp.X1+tmp.X2+tmp.X3+tmp.B1",fmladj1)

+	fml2<-paste(yvname[i],"~xTMP",fmladj1)

+	tmpn<-nrow(wdtmp)

+	print(paste("tp.value ==", tp.value))

+	if (opt=="glm") {

+	tmp.mdl0<-setglm(fml0,i,wdtmp1); tmp.mdl1<-setglm(fml1,i,wdtmp1); tmp.mdl2<-setglm(fml2,i,wdtmp1);

+	}

+	if (opt=="gam") {

+	tmp.mdl0<-setgam(fml0,i,wdtmp1); tmp.mdl1<-setgam(fml1,i,wdtmp1); tmp.mdl2<-setgam(fml2,i,wdtmp1);

+	}

+	if (opt=="gee") {

+	tmp.mdl0<-setgee(fml0,i,wdtmp1); tmp.mdl1<-setgee(fml1,i,wdtmp1); tmp.mdl2<-setgee(fml2,i,wdtmp1);

+	}

+	m2<-mdl2oo(tmp.mdl2,"xTMP",opt)

+	m1<-mdl2oo(tmp.mdl1,c("tmp.X1","tmp.X2","tmp.X3"),opt)

+	m0<-mdl2oo(tmp.mdl0,c("tmp.X1","tmp.X3"),opt)

+	if (opt=="gee") {

+	plrt<-try(anova(tmp.mdl0,tmp.mdl2)$"P(>|Chi|)",TRUE)

+	plrt<-ifelse((plrt<="9" && plrt>="0"), pvformat(plrt,3),"-")

+	} else {plrt<- pvformat(1-pchisq(2*(logLik(tmp.mdl0)[1]-logLik(tmp.mdl2)[1]),1),3);}

+	plrti<-""; plrtx<-""

+	if (!is.na(g) & g==nblv) {

+	fmli<-paste(yvname[i],"~xTMP+xTMP*factor(",bvar,")",fmladj)

+	fmlx<-paste(yvname[i],"~tmp.X1+factor(",bvar,")*tmp.X1+tmp.X2+factor(",bvar,")*tmp.X2+tmp.X3+factor(",bvar,")*tmp.X3+tmp.B1",fmladj)

+	if (opt=="gee") {

+	tmp.mdli<-setgee(fmli,i,wdtmp1)

+	tmp.mdlx<-setgee(fmlx,i,wdtmp1)

+	} else {

+	if (opt=="glm") {tmp.mdli<-setglm(fmli,i,wdtmp1);tmp.mdlx<-setglm(fmlx,i,wdtmp1);}

+	if (opt=="gam") {tmp.mdli<-setgam(fmli,i,wdtmp1);tmp.mdlx<-setgam(fmlx,i,wdtmp1);}

+	plrti<- paste("P-interaction:", pvformat(1-pchisq(2*(logLik(tmp.mdli)[1]-logLik(tmp.mdl2)[1]),nblv-2),3));

+	plrtx<- paste("P-interaction:", pvformat(1-pchisq(2*(logLik(tmp.mdlx)[1]-logLik(tmp.mdl1)[1]),3*(nblv-2)),3));

+	}

+	print(summary(tmp.mdli));

+	print("Interaction model === >")

+	print(paste("tp.value ==", tp.value))

+	print(summary(tmp.mdlx))

+	}

+	oo<-list(c(plrti,m2,plrtx,paste(tp.value,collapse=", "),m1,m0,plrt),tmpn)

+	return(oo)

+ }

> getci4tp<-function(i,j,g,opt,tp0=NA) {

+	set.seed(123456)

+	if (is.na(g)) {wdt<-removeNA(i,j,WD);

+	} else if (g<nblv) {wdt<-WD[WD[,bvar]==blv[g],];wdt<-removeNA(i,j,wdt);

+	} else {wdt<-removeNA(i,j,WD);}

+	nnwd<-nrow(wdt); tp.vv<-rep(NA,1000)

+	if (!is.na(tp0)) {

+	tpp0 = sum(wdt[,xvname[j]] < tp0)/length(wdt[,xvname[j]])

+	tppmin = max(tpp0 - 0.09, 0.05)

+	tppmax = min(tpp0 + 0.09, 0.95)

+	} else {

+	tppmin = NA; tppmax = NA

+	}

+	for (s in (1:1000)) {

+	WDi<-wdt[sample(1:nnwd,nnwd,replace=T),]

+	tp.vv[s]<-get.tpval(i, j, NA, opt, WDi, tppmin, tppmax); rm(WDi)

+	}

+	tpci<-paste(quantile(tp.vv,probs=c(0.025,0.975)),collapse=", ")

+	return(tpci);

+ }

> if (!is.na(weights.var)) {weights<-WD[,weights.var];} else {weights<-1;}

> WD<-cbind(WD,weights);

> vlabelN<-(substr(vlabel,1,1)==" ");

> vlabelZ<-vlabel[vlabelN];vlabelV<-vlabel[!vlabelN]

> vnameV<-vname[!vlabelN];vnameZ<-vname[vlabelN];

> allvname<-c(yvname,xvname,bvar,avname,subjvname,vname.start,timevar,"weights");

> allvname<-allvname[!is.na(allvname)]

> WD<-WD[,allvname];

> w<-c("<!DOCTYPE html><html lang='zh'><head><meta charset='utf-8'></head><body>")

> w<-c(w,paste("<h2>", title, "</h2>"))

> if (length(avname)>0) {

+	if (sum((saf=="s" | saf=="S") & alv>0)>0) w<-c(w,"</br>Spline smoothing only applies for continuous variables")

+	if (!is.na(subjvname)) saf<-rep(0,length(saf))

+ }

> if (sum(xlv>0)>0) w<-c(w,"Categorical exposure variables were ignored")

> xvname<-xvname[xlv==0];

> if (!is.na(subjvname)) WD<-WD[order(WD[,subjvname]),];

>

> fmladj<-""; avb=""; smoothav<-0;

> if (length(avname)>0) {

+	avb<-vlabelV[match(avname,vnameV)];

+	avname_ <- avname

+	smoothavi<-((saf=="s" | saf=="S") & alv==0)

+	smoothav<-sum(smoothavi)

+	avname_[smoothavi]<-paste("s(",avname[smoothavi],")",sep="")

+	avb1<-avb

+	avb1[smoothavi]<-paste(avb[smoothavi],"(Smooth)",sep="")

+	avname_[alv>0]<-paste("factor(",avname[alv>0],")",sep="")

+	fmladj<-paste("+",paste(avname_,collapse="+"))

+ }

> if (is.na(bvar)) {

+	blvb<-"N"; blvb_<-"N"; nblv<-1; blbl<-"";

+ } else {

+	blbl<-vlabelV[match(bvar,vnameV)]; if (is.na(blbl)) blbl<-bvar;

+	blv<-levels(factor(WD[,bvar])); nblv<-length(blv)+1

+	blvb_<-vlabelZ[match(paste(bvar,".",blv,sep=""),vnameZ)];

+	blvb_[is.na(blvb_)]<-blv[is.na(blvb_)];

+	blvb<-c(paste(blbl,blvb_,sep="="),"Total");

+	blvb_<-c(blvb_,"Total")

+	WD<-WD[!is.na(WD[,bvar]),]

+ }

> ny=length(yvname); nx=length(xvname);

> xb<-vlabelV[match(xvname,vnameV)]; xb[is.na(xb)]<-xvname[is.na(xb)]

> yb<-vlabelV[match(yvname,vnameV)]; yb[is.na(yb)]<-yvname[is.na(yb)]

> opt<-ifelse(!is.na(subjvname), "gee", ifelse(smoothav>0, "gam", "glm")) ;

> colprn<-prn

> if (is.na(par1)) par1<-"";

> if (is.numeric(par1)) {tp.vv<-par1;

+ } else {

+	tmp<-as.numeric(strsplit(gsub(",", " ",par1)," ")[[1]]); tp.vv<-c(tmp[!is.na(tmp)],NA)

+ }

> prn<-ifelse(!is.na(bvar), "S", ifelse(nx>1 & ny==1, "X", "Y"));

> if (length(tp.vv)>2) tp.vv<-tp.vv[1:2]

> ntp<-length(tp.vv);

> getci<-FALSE

> prnopt<-c("β (95%CI) Pvalue / OR (95%CI) Pvalue", "β (95%CI) Pvalue", "β+se / OR (95%CI) *P<0.05 **P<0.01 ***P<0.001")

>

>

> if (ntp==1) {

+	cc0<-c("模型 I","&nbsp&nbsp一条直线效应");

+	cc0<-c(cc0,"模型 II","&nbsp&nbsp折点(K)","&nbsp&nbsp &lt K 段效应 1","&nbsp&nbsp &gt K 段效应 2","&nbsp&nbsp 2与1的效应差")

+	cc0<-c(cc0,"&nbsp&nbsp折点处方程预测值")

+	if (is.na(tp.vv[1]) & chk==1) getci<-TRUE;

+ } else {

+	cc0<-c("模型 I","&nbsp&nbsp一条直线效应");

+	cc0<-c(cc0,"模型 II","&nbsp&nbsp折点(K1,K2)","&nbsp&nbsp &lt K1 段效应 1","&nbsp&nbsp K1-K2 段效应 2","&nbsp&nbsp &gt K2 段效应 3")

+	cc0<-c(cc0,"&nbsp&nbsp 1与2的效应差","&nbsp&nbsp 3与2的效应差")

+ }

> if (opt=="gee") {cc0<-c(cc0,"ANOVA 两模型比较");} else {cc0<-c(cc0,"对数似然比检验");}

> if (getci) cc0<-c(cc0,"折点的95可信区间");

> sink(paste(ofname,".lst",sep=""))

> nn<-c("Outcome","Exposure",blvb);

> if (prn=="Y") {

+	for (j in 1:nx) {

+	tt<-cc0;

+	for (i in 1:ny) {

+	if (is.na(tp.vv[1])) {tp.v<-get.tpval(i,j,NA,opt,NA);} else {tp.v<-tp.vv;}

+	if (ntp==1) tmpij<-get2lines(i,j,NA,tp.v,opt);

+	if (ntp==2) tmpij<-get3lines(i,j,NA,tp.v,opt);

+	if (getci) {tt<-cbind(tt,c(tmpij[[1]],getci4tp(i,j,NA,opt,tp.v)));} else {tt<-cbind(tt,tmpij[[1]]);}

+	nn<-rbind(nn,c(yb[i],xb[j],tmpij[[2]]))

+	}

+	tt<-rbind(c("Outcome: ",yb),tt)

+	w<-c(w,paste("</br>For exposure:",xb[j]))

+	w<-c(w,"</br><table border=3>", mat2htmltable(tt), "</table>")

+	}

+ }

> if (prn=="X") {

+	for (i in 1:ny) {

+	tt<-cc0;

+	for (j in 1:nx) {

+	if (is.na(tp.vv[1])) {tp.v<-get.tpval(i,j,NA,opt,NA);} else {tp.v<-tp.vv;}

+	if (ntp==1) tmpij<-get2lines(i,j,NA,tp.v,opt);

+	if (ntp==2) tmpij<-get3lines(i,j,NA,tp.v,opt);

+	if (getci) {tt<-cbind(tt,c(tmpij[[1]],getci4tp(i,j,NA,opt,tp.v)));} else {tt<-cbind(tt,tmpij[[1]]);}

+	nn<-rbind(nn,c(yb[i],xb[j],tmpij[[2]]))

+	}

+	tt<-rbind(c("Exposure: ",xb),tt)

+	w<-c(w,paste("</br>For outcome:",yb[i]))

+	w<-c(w,"</br><table border=3>", mat2htmltable(tt), "</table>")

+	}

+ }

> if (prn=="S") {

+	for (i in 1:ny) {

+	tt<-cc0;

+	for (j in 1:nx) {

+	nnij<-c(yb[i],xb[j])

+	for (g in 1:nblv) {

+	if (is.na(tp.vv[1])) {tp.v<-get.tpval(i,j,g,opt,NA);} else {tp.v<-tp.vv;}

+	if (ntp==1) tmpij<-get2lines(i,j,g,tp.v,opt);

+	if (ntp==2) tmpij<-get3lines(i,j,g,tp.v,opt);

+	if (getci) {tt<-cbind(tt,c(tmpij[[1]],getci4tp(i,j,g,opt,tp.v)));} else {tt<-cbind(tt,tmpij[[1]]);}

+	nnij<-c(nnij,tmpij[[2]])

+	}

+	nn<-rbind(nn,nnij)

+	}

+	tt<-rbind(c(blbl,blvb_),tt)

+	w<-c(w,paste("</br>For outcome:",yb[i]))

+	w<-c(w,paste("</br>For Exposure:",xb[j]))

+	w<-c(w,"</br><table border=3>", mat2htmltable(tt), "</table>")

+	}

+ }

> sink()

> w<-c(w,"</br>表中数据：", prnopt[colprn])

> w<-c(w,paste(c("</br>结果变量:",paste(yb,collapse="; ")),collapse=" "))

> w<-c(w,paste(c("</br>暴露变量:",paste(xb,collapse="; ")),collapse=" "))

> if (length(avname)==0) avb1<-"None";

> w<-c(w,paste(c("</br>调整变量:",paste(avb1,collapse="; ")),collapse=" "))

> if (smoothav>0) w<-c(w,". Generalized additive models were applied")

> if (opt=="gee") w<-c(w, paste("</br>Generalized estimate equation were used, subject ID=", subjvname, "(", gee.TYPE,")",sep=""))

> w<-c(w,"</br></br>各模型所用的样本量</br><table border=3>", mat2htmltable(nn), "</table>")

> w<-c(w,wd.subset)

> w<-c(w,paste("</br>此表用易侕统计软件 (www.empowerstats.com) 和R软件生成，生成日期：",Sys.Date()))

> w<-c(w,"</body></html>")

> fileConn<-file(paste(ofname,".htm",sep="")); writeLines(w, fileConn)

>

>

> proc.time()

user system elapsed

3.42	0.67	11.34
