# Supplementary material for: Association between sleep duration and chronic lung diseases among Chinese middle-aged and older adults: A cross-sectional study
Source: PLoS One. 2026 Mar 19;21(3):e0337482. doi: 10.1371/journal.pone.0337482 (PMC13001977; doi:10.1371/journal.pone.0337482)
Supplement: Attachment — Submitted filename: Response to Reviewers new.docx [file pone.0337482.s006.docx]

We deeply appreciate the consideration of our manuscript by the Journal's Editorial Board. We also thank the reviewer 2 for the comments and suggestions. We have made revisions to the relevant parts of the manuscript. The main modified part was marked in green in the manuscript. The responses to academic editor and the reviewer's comments are as follows:

**Academic editor:**

1. A rebuttal letter that responds to each point raised by the academic editor and reviewer(s). You should upload this letter as a separate file labeled 'Response to Reviewers'.

We greatly appreciate your reminder. The responses have been completed and upload.

2. A marked-up copy of your manuscript that highlights changes made to the original version. You should upload this as a separate file labeled 'Revised Manuscript with Track Changes'.

We greatly appreciate your reminder. A marked-up copy of the manuscript have been completed and upload.

3. An unmarked version of your revised paper without tracked changes. You should upload this as a separate file labeled 'Manuscript'.

We greatly appreciate your reminder. An unmarked version of our revised paper have been completed and upload.

4. If you would like to make changes to your financial disclosure, please include your updated statement in your cover letter.

Thank you very much for your reminder. Financial disclosure did not change.

5. Guidelines for resubmitting your figure files are available below the reviewer comments at the end of this letter.

Thank you very much for your reminder. New figure files have revised and uploaded.

**Reviewer 2:**

**Major Concerns**

**1. Study Design and Causal Inference:**
The cross-sectional design of the study inherently limits the ability to draw causal inferences between sleep duration and chronic lung diseases (CLDs). While the authors acknowledge this limitation, **the discussion** does not sufficiently address how this design might influence the interpretation of the observed associations. Future work employing longitudinal data would be more appropriate for establishing causality.

Thank you very much for raising this important point about study design. We fully acknowledge the inherent limitations of cross-sectional design in establishing causality. Because, it simultaneously measure exposure and outcome variables, and are unable to determine the temporal sequence between them, which raises the possibility of reverse causality. Additionally, the cross-sectional design can not fully account for potential confounders that might bias the observed association.

Similar to other studies (Wang S, Wu Y, Ungvari GS, Ng CH, Forester BP, Gatchel JR, et al. Sleep duration and its association with demographics, lifestyle factors, poor mental health and chronic diseases in older Chinese adults. Psychiat Res. 2017;257:212-8. <http://doi.org/10.1016/j.psychres.2017.07.036>; Ruan Z, Li D, Cheng X, Jin M, Liu Y, Qiu Z, et al. The association between sleep duration, respiratory symptoms, asthma, and COPD in adults. Front Med-Lausanne.2023;10:1108663.<http://doi.org/10.3389/fmed.2023.1108663>; Wang S, Li B, Wu Y, Ungvari GS, Ng CH, Fu Y, et al. Relationship of Sleep Duration with Sociodemographic Characteristics, Lifestyle, Mental Health, and Chronic Diseases in a Large Chinese Adult Population. J Clin Sleep Med. 2017;13(3):377-84. <http://doi.org/10.5664/jcsm.6484>), This cross-sectional study aimed to establish associations rather than causality.

In order to obtain reliable results, we have taken some measures. **Firstly,** statistical methods controled more confounding factors. In addition to common variables such as age and smoking, cooking fuel and depression were also included; **Secondly,** in the sensitivity analysis, the robustness of the results was further verified through subgroup analyses and interaction tests. **Additionally,** we have added subgroup analyses of the remaining variables to further prove the robustness of the results, and they were consistent with our main findings (S3 Table, uploaded it to the submission system in the form of supporting information).

S3 Table Subgroup analysis between sleep duration and chronic lung diseases

| Subgroup | Sleep duration (h) | Adjusted OR (95% CI) | P for interaction |
| --- | --- | --- | --- |
| Marital status |  |  | 0.608 |
| Married/living with partner | 7-8 | Reference |  |
|  | < 7 | 1.34 (1.13, 1.59) |  |
|  | > 8 | 1.21 (0.99, 1.47) |  |
| Other | 7-8 | Reference |  |
|  | < 7 | 1.00 (0.66, 1.50) |  |
|  | > 8 | 1.01 (0.62, 1.63) |  |
| Drinking Status |  |  | 0.451 |
| More than once a month | 7-8 | Reference |  |
|  | < 7 | 1.37 (1.00, 1.89) |  |
|  | > 8 | 1.17 (0.81, 1.68) |  |
| Less than once a month | 7-8 | Reference |  |
|  | < 7 | 0.74 (0.41, 1.32) |  |
|  | > 8 | 0.95 (0.50, 1.80) |  |
| Non-drinker | 7-8 | Reference |  |
|  | < 7 | 1.34 (1.10, 1.63) |  |
|  | > 8 | 1.21 (0.97, 1.52) |  |
| Hypertension |  |  | 0.348 |
| Yes | 7-8 | Reference |  |
|  | < 7 | 1.27 (0.99, 1.63) |  |
|  | > 8 | 1.35 (1.02, 1.78) |  |
| No | 7-8 | Reference |  |
|  | < 7 | 1.30 (1.06, 1.60) |  |
|  | > 8 | 1.06 (0.83, 1.36) |  |
| Diabetes |  |  | 0.417 |
| Yes | 7-8 | Reference |  |
|  | < 7 | 1.61 (1.02, 2.53) |  |
|  | > 8 | 1.05 (0.62, 1.80) |  |
| No | 7-8 | Reference |  |
|  | < 7 | 1.26 (1.06, 1.49) |  |
|  | > 8 | 1.21 (0.99, 1.47) |  |
| Dyslipidemia |  |  | 0.107 |
| Yes | 7-8 | Reference |  |
|  | < 7 | 1.17 (0.88, 1.57) |  |
|  | > 8 | 0.90 (0.63, 1.27) |  |
| No | 7-8 | Reference |  |
|  | < 7 | 1.34 (1.11, 1.62) |  |
|  | > 8 | 1.34 (1.08, 1.66) |  |
| Cardiovascular diseases |  |  | 0.745 |
| Yes | 7-8 | Reference |  |
|  | < 7 | 1.07 (0.75, 1.53) |  |
|  | > 8 | 1.10 (0.72, 1.68) |  |
| No | 7-8 | Reference |  |
|  | < 7 | 1.35 (1.13, 1.61) |  |
|  | > 8 | 1.20 (0.98, 1.48) |  |
| Depression |  |  | 0.238 |
| Yes | 7-8 | Reference |  |
|  | < 7 | 1.17 (0.92, 1.48) |  |
|  | > 8 | 1.20 (0.89, 1.63) |  |
| No | 7-8 | Reference |  |
|  | < 7 | 1.42 (1.15, 1.76) |  |
|  | > 8 | 1.17 (0.93, 1.48) |  |
| Adjusted for all covariates except effect modifier. | | | |

In the limitations, we explained the influence of cross-sectional study on the interpretation of the conclusion. Further, we carefully examine the word of the article, using association rather than causality. The modified content was marked in green in the seventh paragraph of the Discussion section. As shown below:

First, the cross-sectional study can not infer causal relationships. Because, it simultaneously measure exposure and outcome variables, and are unable to determine the temporal sequence between them, which raises the possibility of reverse causality. Additionally, residual confounding by unmeasured variables inevitably exist in cross-sectional studies that may bias the observed association.

**2. Measurement of Key Variables:**
Sleep duration is assessed using self-reported measures that combine nighttime sleep and nap duration. Self-reported data are prone to recall bias and misclassification, potentially impacting the reliability of the exposure measurement. Moreover, the method for categorizing sleep duration (<7, 7–8, and >8 hours) appears somewhat arbitrary and may not capture the nuances of sleep behavior in this population.

Thank you very much for your feedback. We admit that self-reported exposure may have recall bias. For the measurement methods for sleep duration, we referred to the relevant literature (Chen, X., et al., Relationship between sleep duration and sociodemographic characteristics, mental health and chronic diseases in individuals aged from 18 to 85 years old in Guangdong province in China: a population-based cross-sectional study. BMC Psychiatry, 2020. 20(1): p. 455. Wang, S., et al., Sleep duration and its association with demographics, lifestyle factors, poor mental health and chronic diseases in older Chinese adults. Psychiatry Research, 2017. 257: p. 212-218). Additionaly, studies found that subjectively reported sleep duration modestly correlated with objectively sleep duration sleep duration (Hoch, C.C., Reynolds, C.F., Kupfer, D.J., Berman, S.R., Houck, P.R., Stack, J.A., 1987. Empirical note: self-report versus recorded sleep in healthy seniors. Psychophysiology 24, 293–299). We excluded some participants with mental illness or memory illness to increase the information accuracy. These may increase the reliability of the self-reported exposure measurement.

The classification method of sleep duration also refers to some literatures (Wang S, Wu Y, Ungvari GS, Ng CH, Forester BP, Gatchel JR, et al. Sleep duration and its association with demographics, lifestyle factors, poor mental health and chronic diseases in older Chinese adults. Psychiat Res. 2017;257:212-8. <http://doi.org/10.1016/j.psychres.2017.07.036>). In multiple regression models (Table 2, marked in green), we analyzed sleep duration as a continuous variable, for every additional hour, the risk of CLDs increases by 4% (OR 0.96, 95% CI 0.93-0.99) adjusted for all covariates. This may refine the degree of influence of sleep duration on CLDs.

| Table 2 Logistic regression analysis between sleep duration and chronic lung diseases. | | | | | | |
| --- | --- | --- | --- | --- | --- | --- |
| Variables | Model 1 | P | Model 2 | P | Model 3 | P |
|  | OR (95% CI) |  | OR (95% CI) |  | OR (95% CI) |  |
| Sleep duration (h) | 0.92 (0.89, 0.95) | < 0.001 | 0.93 (0.90, 0.96) | < 0.001 | 0.96 (0.93, 0.99) | 0.004 |
| Sleep duration (h) |  |  |  |  |  |  |
| 7-8 | Reference |  | Reference |  | Reference |  |
| < 7 | 1.52 (1.31, 1.78) | < 0.001 | 1.44 (1.23, 1.69) | < 0.001 | 1.29 (1.10, 1.52) | 0.002 |
| > 8 | 1.26 (1.05, 1.51) | 0.012 | 1.18 (0.99, 1.42) | 0.072 | 1.18 (0.98, 1.42) | 0.072 |
| Note: Model 1 adjusted for nothing. Model 2 adjusted for age, gender, marital status, education level, residence region, BMI, smoke, drink. Model 3 adjusted for model 2 plus cooking fuel, hypertension, diabetes, dyslipidemia, cardiovascular disease and depression. | | | | | |  |
| In the limitations, we explained the influence of self-reported sleep duration on the reliability of the exposure measurement. The modified content was marked in green in the seventh paragraph of the Discussion section. As shown below:  Second, sleep duration measures in our study were self-reported by participants and were subject to recall bias. Although subjectively reported sleep duration modestly correlated with objectively sleep duration [43], more precise techniques such as polysomnography are still needed for future study. | | | | | | |
|  | | | | | | |

**3. Statistical Analysis and Interpretation:**
The manuscript uses multiple logistic regression models and a three-piecewise linear regression to explore nonlinearity. However, the rationale for selecting the inflection points at 7 and 8 hours is not convincingly justified by the data or by previous literature. There are also concerns regarding the handling of missing data (using deletion for some variables and dummy coding for others), which might lead to residual confounding. The interpretation of non-significant findings for long sleep duration is ambiguous, and alternative explanations for the observed associations are not fully explored.

Thank you very much for your detailed observation.

**For three-piecewise linear regression:** Representing it with a three-piecewise linear regression is not very accurate. We referred to several articles and modified it to the two-piecewise regression model (Zhou M, Cai H, Yi W, Gao X. A Nonlinear Relationship Between ALT Levels at Delivery and the Risk of Postpartum ALT Flares in Pregnant Women with Chronic Hepatitis B. Int J Med Sci. 2023;20(2):247-253. doi: 10.7150/ijms.79663; Chen, C, Dai JL. Triglyceride to high-density lipoprotein cholesterol (HDL-C) ratio and arterial stiffness in Japanese population: a secondary analysis based on a cross-sectional study. Lipids Health Dis, 2018. 17(1): p. 130.).

The original text has also been revised in the second paragraph of the Statistical analysis (Methord section), marked in green. As shown below:

Threshold effect analysis was conducted using the two-piecewise regression model and the log likelihood ratio test

**For selecting the inflection points at 7 and 8 hours:** The selection of inflection points is based on the curve fitting graph presented by the data and combined with some literatures. As shown in the following Fig 2, the inflection points at 7 and 8 hours may exist, and log likelihood ratio test comparing one-line (non-segmented) model to segmented regression model also indicated that the threshold existed (S2 Table). In addition, the multiple literatures (Ruan Z, Li D, Cheng X, Jin M, Liu Y, Qiu Z, et al. The association between sleep duration, respiratory symptoms, asthma, and COPD in adults. Front Med-Lausanne. 2023;10:1108663. <http://doi.org/10.3389/fmed.2023.1108663>) have also obtained similar results, and the inflection point is around 7.5. However, considering that our study population consists of middle-aged and elderly Chinese individuals, extrapolation beyond the inflection point should be approached with caution.

We have addressed these points in the limitations part, located in the seventh paragraph of the Discussion section, marked in green. As shown below:

Lastly, our study conducted in participants aged ≥ 45 years in China, this findings do not generalize to those younger than 45 years of age or other countries.

| S2 Table Threshold effect analysis for the relationship between sleep duration and CLDs | | |
| --- | --- | --- |
| CLDs | Adjusted OR (95% CI) | P value |
| Linear regression model | 0.96 (0.93, 0.99) | 0.005 |
| Two-piecewise linear regression model |  |  |
| Inflection point | 7, 8 |  |
| Sleep duration (h) < 7 | 0.92 (0.87, 0.98) | 0.006 |
| Sleep duration (h) 7-8 | 0.85 (0.68, 1.07) | 0.160 |
| Sleep duration (h) > 8 | 0.91 (0.80, 1.04) | 0.156 |
| Log likelihood ratio test |  | 0.002 |
| CLDs: chronic lung diseases. Adjusted for all covariates. | | |

**For the missing data:** We calculated the number of missing cases and performed multiple imputation. The specific results can be found in the Supporting information (S4 Table, S5 Table). Analysis of the imputed data yielded consistent results, indicating that shorter sleep duration was associated with a higher prevalence of CLDs, and the turning points at 7 and 8 hours remained exist.

The original text has also been revised in the second paragraph of the Statistical analysis (Methord section), in the second paragraph of the Sensitivity analysis (Resulds section), marked in green. As shown below:

| and multivariate logistic regression analysis after multiple imputation for missing data.  In addition, the results of multivariate regression analysis after multiple imputation for missing data showed short sleep duration was associated with CLDs (OR 1.30, 95% CI 1.11-1.51) (S4 Table). A non-linear relationship between sleep duration and CLDs was also found in the dataset of multiple imputation for missing data. The inflection points of 7 and 8 hours remained exist (S5 Table).  S4 Table. Logistic regression analysis between sleep duration and chronic lung diseases. | | | | | | | | | |  |
| --- | --- | --- | --- | --- | --- | --- | --- | --- | --- | --- |
| Variables | Model 1 | P | Model 2 | | P | Model 3 | | | P |  |
|  | OR (95% CI) |  | OR (95% CI) | |  | OR (95% CI) | | |  |  |
| Sleep duration | 0.93 (0.90, 0.95) | < 0.001 | 0.93 (0.91, 0.96) | | < 0.001 | 0.96 (0.93, 0.99) | | | 0.015 |  |
| Sleep duration |  |  |  | |  |  | | |  |  |
| 7-8 | Reference |  | Reference | |  | Reference | | |  |  |
| < 7 | 1.53 (1.32, 1.78) | < 0.001 | 1.45 (1.24, 1.69) | | < 0.001 | 1.30 (1.11, 1.51) | | | 0.001 |  |
| > 8 | 1.31 (1.10, 1.56) | 0.003 | 1.23 (1.03, 1.46) | | 0.024 | 1.23 (1.03, 1.47) | | | 0.023 |  |
| Model 1 adjusted for nothing. | | | | | | | | |  |  |
| Model 2 adjusted for age, gender, marital status, education, residence region. | | | | | | | | | | |
| Model 3 adjusted for model 2 plus BMI, smoke, drink, cooking fuel, hypertension, diabetes, dyslipidemia, cardiovascular disease and depression. | | | | | | | | | |  |
| S5 Table. Threshold effect analysis for the relationship between sleep duration and CLDs. | | | | | | | |  |  |  |
| CLDs | | | | Adjusted OR (95% CI) | | | P |  |  |  |
| Linear regression model | | | | 0.96 (0.93, 0.99) | | | 0.015 |  |  |  |
| Two-piecewise linear regression model | | | |  | | |  |  |  |  |
| Inflection point | | | | 7, 8 | | |  |  |  |  |
| Sleep duration (h) < 7 | | | | 0.93 (0.88, 0.98) | | | 0.012 |  |  |  |
| Sleep duration (h) 7-8 | | | | 0.85 (0.68, 1.06) | | | 0.150 |  |  |  |
| Sleep duration (h) > 8 | | | | 0.89 (0.79, 1.01) | | | 0.081 |  |  |  |
| Log likelihood ratio test | | | |  | | | < 0.001 |  |  |  |
| CLDs: chronic lung diseases. Adjusted for all covariates. | | | | | | | |  |  |  |

**For the non-significant findings for long sleep duration** Our results revealed that there was no significant statistical association between long sleep and CLDs. Because, the sample size of the group with more than 8 hours of sleep is relatively small, which may have contributed to the lack of positive results. Further studies with larger samples are necessary. We have revised the results section of the Abstract and removed the negative results for the group with more than 8 hours of sleep.

**4. Novelty and Contribution to the Field:**
While the use of a large dataset is a strength, the overall findings do not present substantial novelty relative to the existing literature on sleep and respiratory health. Several previous studies have reported similar associations or conflicting results. The manuscript would benefit from a more robust discussion that clearly situates its contributions against the current state of research.

Thank you very much for your suggestion. As you mentioned, we have thoroughly discussed the reasons for the differences in findings across studies in the fifth paragraph of the Discussion section, and strengths in the seventh paragraph of the Discussion section, marked in green. As shown below:

These inconsistent findings across studies can be explained by several critical factors. First, the representativeness of the studied population must be considered. Demographic differences, including variations in ethnicity and age distribution, could potentially influence the study results. Second, different studies used varying methods to define sleep duration and categorize it as insufficient, normal, or excessive, which may affect the results. Additionally, whether the duration of sleep includes napping time may also influence the results. Finally, the degree of control for potential confounders also may led to differences. It is important to recognize that all studies did not uniformly adjust for factors such as socioeconomic status, underlying health conditions, or psychological variables. Our research specifically adjusted for several pertinent factors, such as cooking fuel types and depression, which may influence respiratory health outcomes and sleep duration

This study had several strengths. Our research originated from high-quality national cohort study, and included and controlled for a considerable number of possible confounding factors such as cooking fuel and depression. Additionally, the consistency of more subgroup analyses and interaction tests indicated the robustness of our results. Our study explored the association between total sleep duration within a 24-hour period and CLDs, and excluded the potential influence of nap time on nighttime sleep.

**Minor Concerns

1. Clarity and Writing:**
The manuscript contains numerous typographical and grammatical errors that detract from the overall clarity. A thorough revision and copy-editing are recommended to enhance readability and ensure the arguments are presented in a clear, concise manner.
Thank you very much for your feedback. We have carefully carried out the typographical and grammatical checks and uploaded the revised manuscript.

**2. Presentation of Data**:
Some figures and tables are not well integrated into the narrative. For example, the descriptions of Tables 1–4 and Figure 2 lack sufficient detail in the text. Clear labeling and more comprehensive explanations of the results would improve the presentation.
Thank you very much for your suggestion. We have provided more comprehensive explanations for the Tables and Figures in the Results section, marked in green, and added the labels for Figures. As shown below:

As demonstrated in Table 1, 5,773 (42.0%) individuals slept less than 7 hours, 4,711 (34.2%) slept between 7-8 hours, and 3,275 (25.8%) slept more than 8 hours. Age, gender, marital status, residence region, education level, smoking status, drinking status, BMI, cooking fuel, hypertension, cardiovascular diseases, and depression all exhibited statistically significant differences across the groups (P < 0.05). While, diabetes and dyslipidemia were not statistically significant across the groups (P > 0.05). Participants in the short sleep duration group were more likely to have CLDs (8.5%), and CLDs was statistically significant across the groups (P < 0.001).

Table 2 illustrated the association between sleep duration and CLDs. When treated as continuous variable, each additional hour of sleep was associated with a 8% decrease in the prevalence of CLDs (OR 0.92, 95% CI 0.89-0.95) in the model 1; each additional hour of sleep was associated with a 7% decrease in the prevalence of CLDs (OR 0.93, 95% CI 0.90-0.96) in the model 2; each additional hour of sleep was associated with a 4% decrease in the prevalence of CLDs (OR 0.96, 95% CI 0.93-0.99) in the model 3 adjusted for all covariates. As categorical variable, with the reference of normal sleep duration, short sleep duration was positively associated with CLDs (model 1: OR 1.52, 95% CI 1.31-1.78; model 2: OR 1.44, 95% CI 1.23-1.69; model 3: OR 1.29, 95% CI 1.10-1.52).

A non-linear relationship between sleep duration and CLDs was depicted in Figure 2, S1 Figure. With an increase in sleep duration, the probability of CLDs decreased, plateauing at approximately 7-8 hours of sleep, where the probability of CLDs no longer changed.

**3. Ethical and Data Considerations:**
Although the manuscript states that the study received ethical approval from the relevant committee, there is limited discussion on the limitations inherent in using self-reported diagnostic information for CLDs. Addressing how potential misclassification might affect the study’s conclusions would be beneficial.
Thank you very much for your valuable question. We referred to other literatures regarding the definition of CLDs (Wang, Y., et al., Effects and differences of sleep duration on the risk of new-onset chronic disease conditions in middle-aged and elderly populations. European Journal of Internal Medicine, 2023. 107: p. 73-80. Yang, K. and R. Chen, Association between cooking fuel exposure and respiratory health: Longitudinal evidence from the China Health and Retirement Longitudinal Study (CHARLS). Ecotoxicology and Environmental Safety, 2024. 275: p. 116247), but self-reported physician diagnosis may involve recall bias and is a limitation of the article. We have further elaborated on this in the seventh paragraph of the Discussion section. Marked in green. As shown below:

Third, CLDs are assessed through self-reported physician diagnosis without verification from medical records or diagnostic tests. This approach might introduce the potential for misclassification bias. Future studies should incorporate objective measures to improve the accuracy and reliability of disease diagnosis.

**4. Formatting and Reference Consistency:**
Several references and in-text citations appear inconsistent. Please ensure that all references are correctly formatted and that the citations support the claims made in the text.
Thank you very much for your feedback. We carefully checked the consistency between the content of the article and the references, and made the necessary revisions. According to the guidelines for citing references, the format of the references has been revised.
