# Supplementary material for: Association between sleep duration and chronic lung diseases among Chinese middle-aged and older adults: A cross-sectional study
Source: PLoS One. 2026 Mar 19;21(3):e0337482. doi: 10.1371/journal.pone.0337482 (PMC13001977; doi:10.1371/journal.pone.0337482)
Supplement: Attachment — Submitted filename: Comments_CLD_revision3.docx [file pone.0337482.s007.docx]

**Statistical Analysis**

The statistical analysis section is confusing and lacks transparency regarding both the analytical flow and rationale for variable inclusion. It is unclear whether the univariate comparisons (ANOVA, Kruskal–Wallis, Chi-square, Fisher’s exact tests) were used only for descriptive purposes or as a basis for variable selection. If the latter, this approach is inappropriate, variables for adjustment should not be selected automatically based on statistical significance. Instead, covariate selection should rely on theoretical justification, prior evidence, or known confounders.

The handling of missing data is also insufficiently described. The authors should clearly specify the number of imputations performed, the imputation model used (including which variables were included), and whether results were pooled following Rubin’s rules. The reference to “multivariate logistic regression” is incorrect and should be “multivariable logistic regression,” as only one outcome was modeled with multiple predictors.

I recommend the authors restructure the statistical analysis section to present a coherent and logical workflow: (1) descriptive and univariate analyses for baseline characteristics; (2) model specification with explicit justification for covariate inclusion; (3) assessment of nonlinearity and model assumptions; (4) sensitivity and subgroup analyses with rationale. Each step should be described clearly, avoiding data-driven or stepwise selection procedures, and ensuring that model choices are grounded in substantive reasoning rather than significance testing.

Finally, I strongly recommend to avoid unnecessary and irrelevant software outputs. The authors should stick to presenting important results to answer the research questions. It is not entirely clear why the authors given emphasis on the variable selection based on automatic approach which will definitely avoid the true association and lead to bias. Presenting those methods and the results from them could also not be the main purpose of the analysis. I believe addressing these comments will correct the results and the paper will be beneficial in delivering what it intended for.
