# Supplementary material for: Association between sleep duration and chronic lung diseases among Chinese middle-aged and older adults: A cross-sectional study
Source: PLoS One. 2026 Mar 19;21(3):e0337482. doi: 10.1371/journal.pone.0337482 (PMC13001977; doi:10.1371/journal.pone.0337482)
Supplement: Attachment — Submitted filename: Response_to_Reviewers_auresp_3.docx [file pone.0337482.s008.docx]

We sincerely appreciate the editorial department’s diligent efforts on our manuscript and are also grateful for the reviewers’ suggestions. Based on the suggestions, we have made revisions to the relevant parts of the manuscript. The main corrections in the paper and the response to the reviewer’s comments are as follows.

**Statistical Analysis**

1.The statistical analysis section is confusing and lacks transparency regarding both the analytical flow and rationale for variable inclusion. It is unclear whether the univariate comparisons (ANOVA, Kruskal–Wallis, Chi-square, Fisher’s exact tests) were used only for descriptive purposes or as a basis for variable selection. If the latter, this approach is inappropriate, variables for adjustment should not be selected automatically based on statistical significance. Instead, covariate selection should rely on theoretical justification, prior evidence, or known confounders.

Thank you very much for your question. The purpose of using these statistical tests is to compare differences between groups (< 7 hours, 7-8 hours, > 8 hours) and identify factors that may be related to the independent variable, i.e., potential confounding factors, in preparation for variable adjustment. The variables we adjusted for in the models were selected based on the following considerations: prior literature (**5.** Wang S, Wu Y, Ungvari GS, Ng CH, Forester BP, Gatchel JR, et al. Sleep duration and its association with demographics, lifestyle factors, poor mental health and chronic diseases in older Chinese adults. Psychiatry Res. 2017;257:212-218. doi: 10.1016/j.psychres.2017.07.036. 8. Ruan Z, Li D, Cheng X, Jin M, Liu Y, Qiu Z, et al. The association between sleep duration, respiratory symptoms, asthma, and COPD in adults. Front Med (Lausanne). 2023;10:1108663. doi: 10.3389/fmed.2023.1108663. 20. Wang S, Li B, Wu Y, Ungvari GS, Ng CH, Fu Y, et al. Relationship of Sleep Duration with Sociodemographic Characteristics, Lifestyle, Mental Health, and Chronic Diseases in a Large Chinese Adult Population. J Clin Sleep Med. 2017;13(3):377-384. doi: 10.5664/jcsm.6484. 25. Chen X, Wang S, Li X, Huang Z, Tan W, Lin H, et al. Relationship between sleep duration and sociodemographic characteristics, mental health and chronic diseases in individuals aged from 18 to 85 years old in Guangdong province in China: a population-based cross-sectional study. BMC Psychiatry. 2020;20(1):455. doi: 10.1186/s12888-020-02866-9. 26. Yang K, Chen R. Association between cooking fuel exposure and respiratory health: Longitudinal evidence from the China Health and Retirement Longitudinal Study (CHARLS). Ecotoxicol Environ Saf. 2024;275:116247. doi: 10.1016/j.ecoenv.2024.116247), clinical significance, and statistical results.

To clarify this part, we have revised the text as follows:

**The first paragraph of Statistical analysis section:** Continuous variables were expressed as the mean ± standard deviation. Categorical variables were presented as the frequency (percentages). The differences between the groups (< 7 hours, 7-8 hours, > 8 hours) were compared using analysis of variance (for normal distribution) or Kruskal Wallis test (for skewed distribution) for continuous variables, and Chi-square test or Fisher exact test for categorical variables.

**The second paragraph of Statistical analysis section:** Model 3 was adjusted for all covariates. The selection of adjusted variables was primarily based on three key considerations: prior literature[5,8,20,25,26], clinical significance, and statistical results.

2.The handling of missing data is also insufficiently described. The authors should clearly specify the number of imputations performed, the imputation model used (including which variables were included), and whether results were pooled following Rubin’s rules. The reference to “multivariate logistic regression” is incorrect and should be “multivariable logistic regression,” as only one outcome was modeled with multiple predictors.

Thank you for your feedback. All “multivariate logistic regression” have been changed to “multivariable logistic regression.

For missing data: after applying the inclusion and exclusion criteria, BMI data were found to be missing in a significant proportion of cases (16.03%). To preserve a larger sample size, we handled the missing values using dummy variables. We have revised the text as follows:

**The fourth paragraph of Statistical analysis section:** The missing data for BMI was16.03%, and we used dummy variable to indicate missing values. The missing samples were recorded as a group and marked as “unclear”.

Additionally, we performed multiple imputation for the missing data. For the purpose of sensitivity analysis, we simplified the multiple imputation process by including all variables in the model and generating only one imputed dataset, thus no pooling was performed. We conducted all primary analyses on this dataset, including multiple regression equations, curve fitting, and threshold effect analysis. The results were consistent across these analyses and aligned with those from the main analysis. We acknowledge that this may not be rigorous, so, to further verify the robustness of the results, we conducted an analysis using the MICE package in R: Five imputations were performed with 50 iterations, using LOGREG for binary variables and POLYREG for unordered categorical variables, with all variables included in the imputation process. The results remained stable in the full adjusted model, as shown in the figure below and S3 Table.

We have revised the text as follows:

**The third paragraph of Statistical analysis section:** (2) repeating main regression analyses after the multiple imputation method (based on 5 replications in the R MICE procedure) for addressing missing variables.

3.I recommend the authors restructure the statistical analysis section to present a coherent and logical workflow: (1) descriptive and univariate analyses for baseline characteristics; (2) model specification with explicit justification for covariate inclusion; (3) assessment of nonlinearity and model assumptions; (4) sensitivity and subgroup analyses with rationale. Each step should be described clearly, avoiding data-driven or stepwise selection procedures, and ensuring that model choices are grounded in substantive reasoning rather than significance testing.

Thank you very much for your suggestion. We have revised this section accordingly, as follows:

Continuous variables were expressed as the mean ± standard deviation. Categorical variables were presented as the frequency (percentages). The differences between the groups (< 7 hours, 7-8 hours, > 8 hours) were compared using analysis of variance (for normal distribution) or Kruskal Wallis test (for skewed distribution) for continuous variables, and Chi-square test or Fisher exact test for categorical variables.

Multicollinearity among the variables was assessed using the variance inflation factor (VIF), and those with VIF value exceeding 10 were excluded as they were deemed collinear [33]. Univariate logistic regression model was used to examine the association between variables and CLDs.To investigate the association of sleep duration with CLDs, we employed three logistic regression models. Model 1 was unadjusted. Model 2 was adjusted for age, gender, marital status, residence region, education level, BMI, smoke, drink. Model 3 was adjusted for all covariates. The selection of adjusted variables was primarily based on three key considerations: prior literature[5,8,20,25,26], clinical significance, and statistical results. Generalized additive model with smoothing curve fitting were used to explore potenial nonlinear relationship. Threshold effect analysis was conducted using the two-piecewise regression model and the log likelihood ratio test [34,35].

Sensitivity analysis was performed as follows: (1) subgroup analyses were conducted using logistic regression model adjusting for different covariates except effect modifier to exploratorily examine whether the association between sleep duration and CLDs was moderated by the following characteristics: age, gender, educational level, residence region, BMI, smoking status, cooking fuel. Interaction tests were evaluated using likelihood ratio test. (2) repeating main regression analyses after the multiple imputation method (based on 5 replications in the R MICE procedure) for addressing missing variables.

The missing data for BMI was16.03%, and we used dummy variable to indicate missing values. The missing samples were recorded as a group and marked as “unclear”.

4.Finally, I strongly recommend to avoid unnecessary and irrelevant software outputs. The authors should stick to presenting important results to answer the research questions. It is not entirely clear why the authors given emphasis on the variable selection based on automatic approach which will definitely avoid the true association and lead to bias. Presenting those methods and the results from them could also not be the main purpose of the analysis. I believe addressing these comments will correct the results and the paper will be beneficial in delivering what it intended for.

Thank you very much for your suggestion. Focusing on the research questions to be addressed, we have streamlined our results by removing some unnecessary tables and figures.The following figures and tables have been retained:

Fig 1. Flow chart.

Figure 2 The nonlinear association between CLDs and sleep duration. Solid black line represents the smooth curve fit between variables. Dotted lines bands represent the 95% of confidence interval adjusted for all covariates. CLDs: chronic lung diseases

Table 1. Basic characteristics of participants.

Table 2. Logistic regression analysis between sleep duration and chronic lung diseases.

Table 3. Subgroup analysis between sleep duration and chronic lung diseases.

S1 Table Results of univariate analysis

S2 Table Threshold effect analysis for the relationship between sleep duration and CLDs

S3 Table Logistic regression analysis between sleep duration and chronic lung diseases after multiple imputation
