# Supplementary material for: Association between sleep duration and chronic lung diseases among Chinese middle-aged and older adults: A cross-sectional study
Source: PLoS One. 2026 Mar 19;21(3):e0337482. doi: 10.1371/journal.pone.0337482 (PMC13001977; doi:10.1371/journal.pone.0337482)
Supplement: Attachment — Submitted filename: Response_to_Reviewers_auresp_4.docx [file pone.0337482.s009.docx]

We sincerely appreciate the suggestions and comments from the academic editor and the reviewer. Based on the comments, we have made revisions to the relevant parts of the manuscript. The response and the main corrections in the paper are as follows.

**Journal Requirements:**

If the reviewer comments include a recommendation to cite specific previously published works, please review and evaluate these publications to determine whether they are relevant and should be cited. There is no requirement to cite these works unless the editor has indicated otherwise.

Thank you very much for your reminder. The reviewer did not suggest any citations.

Please review your reference list to ensure that it is complete and correct. If you have cited papers that have been retracted, please include the rationale for doing so in the manuscript text, or remove these references and replace them with relevant current references. Any changes to the reference list should be mentioned in the rebuttal letter that accompanies your revised manuscript. If you need to cite a retracted article, indicate the article’s retracted status in the References list and also include a citation and full reference for the retraction notice.

Thank you very much for your suggestions. All references have been checked, and no retracted articles were cited. Additionally, we identified minor issues in four references, which have been corrected in the manuscript and highlighted in yellow. The revisions are as follows:

**4.** Long Z, Liu W, Qi J, Liu Y, Liu J, You J, et al. Mortality trend of chronic respiratory diseases in China, 1990-2019. Zhonghua Liu Xing Bing Xue Za Zhi. 2022;43(1):14-21. doi: 10.3760/cma.j.cn112338-20210601-00443.

**21**. Yang M, Fan X, Su J, Wan X, Yu H, Lu Y, et al. A prospective study on association between sleep duration and the risk of chronic obstructive pulmonary disease in adults in Suzhou. Zhonghua Liu Xing Bing Xue Za Zhi. 2024;45(3):331-338. doi: 10.3760/cma.j.cn112338-20230918-00164.

**31**. Joint committee for guideline revision. 2016 Chinese guidelines for the management of dyslipidemia in adults. J Geriatr Cardiol. 2018;15(1):1-29. doi: 10.11909/j.issn.1671-5411.2018.01.011.

**43**. Hoch CC, Reynolds CF 3rd, Kupfer DJ, Berman SR, Houck PR, Stack JA. Empirical note: self-report versus recorded sleep in healthy seniors. Psychophysiology. 1987;24(3):293-9. doi: 10.1111/j.1469-8986.1987.tb00298.x.

**Reviewer #3:**

This manuscript investigates the association between sleep duration and chronic lung diseases (CLDs) using data from the 2011 baseline survey of the China Health and Retirement Longitudinal Study (CHARLS). The study addresses an important public health question using a large, nationally representative dataset. The sample size is substantial (n = 13,759), and the statistical methods are generally appropriate.
However, several methodological, analytical, and reporting issues should be addressed before the manuscript is suitable for publication.

1. Study Design and Causal Language: The cross-sectional nature of the study limits causal inference. While the authors acknowledge this limitation, parts of the discussion imply directional interpretation. Revise the discussion to avoid causal language

Thank you very much for your valuable suggestions. We have reviewed the content of the Discussion section and removed the explanations regarding causality, such as terms related to risk and prevention in the sixth paragraph of the Discussion section. The revisions are highlighted in green in the manuscript, as follows:

Our study provided a reference for incorporating sleep management into the management of CLDs, highlighting the need for public health professionals to pay greater attention to sleep health in middle-aged and elderly population. We suggest the following measures to improve sleep health: raising awareness of sleep health through community-based sleep hygiene education programs, conducting sleep problem screening in primary healthcare institutions, and establishing multidisciplinary sleep clinics. Potential barriers may include limited healthcare resources and socio-cultural attitudes toward sleep. We recommend establishing a collaborative mechanism among healthcare providers, policymakers, and community organizations, and modifying socio-economic and cultural norms to promote sleep health.

Additionally, we also remove this sentence (This study supports integrating sleep management into CLDs prevention strategies for middle-aged and elderly population) from **the Conclusion** to eliminate any causal description.

2. CHARLS uses a multistage probability sampling design. The manuscript does not clearly state whether: Sampling weights were applied. Clarify whether survey-weighted logistic regression was used.

Thank you very much for your insightful comments. We fully agree that when using nationally representative survey data with complex sampling designs, such as CHARLS, the correct application of sampling weights and consideration of the survey design are crucial for obtaining descriptive statistics that are representative of the population.

We sincerely acknowledge that in the **association analysis** of this study, sampling weights were not used. This methodological choice was based on our judgment that this study is an **analytical study** rather than a **descriptive study**. Our primary study objective was to explore the **association** between sleep duration and chronic lung diseases, rather than to precisely estimate descriptive parameters such as prevalence in the target population. Many published studies conducting similar association analyses have adopted the same approach of not incorporating sampling weights based on CHARLS (23. Wang Y, Jiang G, Hou N, Chen M, Yang K, Wen K, et al. Effects and differences of sleep duration on the risk of new-onset chronic disease conditions in middle-aged and elderly populations. Eur J Intern Med. 2023;107:73-80. doi: 10.1016/j.ejim.2022.11.005. 33.Song Y, Liu H, Liu Y. The association between nap time, nighttime sleep and depression in Chinese older adults: A cross-sectional study. PLoS One. 2024;19(6):e0302939. doi: 10.1371/journal.pone.0302939.)

In accordance with your valuable suggestions, we provided clarifications in the **Methods** sections, as follow:

**In the Methods section:** This cross-sectional study was an analytical study focusing on associations, not a descriptive study to obtain population estimates; therefore, sampling weights were not used.

Additionally, we have revised the term “nationally representative.” When describing the sample source, we noted that the data originate from a nationally representative survey (CHARLS). However, we avoided using the term “nationally representative” in our study. The term “nationally representative” has been removed from the following sections. The last paragraph of the Introduction, and the first paragraph of the Discussion.

Meanwhile, we have revised the term “prevalence” from the following sections.

**In the first paragraph of the Discussion section:** In this cross-sectional study, short sleep duration (< 7 hours) was associated with a higher **likelihood** of CLDs (OR 1.29, 95% CI 1.10-1.52).

**In the second paragraph of the Discussion section:** Our findings further provided epidemiological evidence that short sleep duration was associated with a higher **likelihood** of CLDs.

3. The manuscript adjusts for hypertension, cardiovascular diseases, diabetes, and depression. Some of these variables may lie on the causal pathway between sleep duration and CLDs, leading to possible overadjustment.

Thank you very much for your insightful comments. In the fully adjusted regression model, the adjustment for these variables was primarily based on the following references:

**5.** Wang S, Wu Y, Ungvari GS, Ng CH, Forester BP, Gatchel JR, et al. Sleep duration and its association with demographics, lifestyle factors, poor mental health and chronic diseases in older Chinese adults. Psychiatry Res. 2017;257:212-218. doi: 10.1016/j.psychres.2017.07.036.

**8.** Ruan Z, Li D, Cheng X, Jin M, Liu Y, Qiu Z, et al. The association between sleep duration, respiratory symptoms, asthma, and COPD in adults. Front Med (Lausanne). 2023;10:1108663. doi: 10.3389/fmed.2023.1108663.

**23.** Wang Y, Jiang G, Hou N, Chen M, Yang K, Wen K, et al. Effects and differences of sleep duration on the risk of new-onset chronic disease conditions in middle-aged and elderly populations. Eur J Intern Med. 2023;107:73-80. doi: 10.1016/j.ejim.2022.11.005.

In addition, considering that these diseases may be mediating variables in the causal pathway between sleep duration and chronic lung disease, we re-conducted the primary regression analysis. The fully adjusted regression model did not include these variables, and the results showed that short sleep duration remained positively associated with chronic lung disease, with an increased odds ratio (OR:1.43, 95%CI:1.22-1.67). The results are presented in the figure below. This suggests that these diseases, sleep duration, and chronic lung disease may have complex interrelationships. This result further demonstrates the robustness of our conclusion.

4. Replace “risk” with “odds” throughout the manuscript.

Thank you very much for your suggestions. All instances of the word “risk” have been replaced with “odds”. Additionally, in the citation for the cross-sectional study, the word “risk” has also been replaced. The revisions are highlighted in green, as follows:

The third paragraph of the Introduction:

Similarly, research from Jilin Province, China indicated an association between limited sleep and increased COPD odds

Another study of non-institutionalized Americans adults showed that both short and long sleep duration were associated with a higher odds of COPD

5. Clarify the calculation of sleep duration (conversion of nap minutes to hours).

Thank you very much for your suggestion. To more clearly express the calculation of sleep duration, we have revised the relevant sections, which are highlighted in green, as follows:

Nighttime sleep duration was assessed based on the question: “During the past month, how many hours of actual sleep did you get at night (average hours for one night)?”. Nap time was assessed based on the question: “During the past month, how long did you take a nap after lunch (minutes)?”. Sleep duration was calculated by adding nighttime sleep duration to nap time, with the nap time first divided by 60 to convert it into hours.
